# Risk prediction models for lymph node metastasis in early gastric cancer patients: a systematic review and meta-analysis

**DOI:** 10.1186/s12876-025-04342-8

**Published:** 2025-10-31

**Authors:** Meng Duan, Min Li, Lei He, Shiming Dai, Lang Zhou, Zhiqun Liu, Jie Yang, Lingyuan Chen, Xiang Liu, Zhaoshu Wu

**Affiliations:** 1 Department of Anorectal Center, Nanjing Hospital of Chinese Medicine Affiliated to Nanjing University of Chinese Medicine, Nanjing, 210022 China; 2 Department of General Surgery, Nanjing Hospital of Chinese Medicine affiliated to Nanjing University of Chinese Medicine, Nanjing, 210000 China; 3 Department of Gastroenterology, Nanjing Hospital of Chinese Medicine Affiliated to Nanjing University of Chinese Medicine, Nanjing, 210000 China; 4 Department of Endoscopy Center, Nanjing Hospital of Chinese Medicine Affiliated to Nanjing University of Chinese Medicine, Nanjing, 210000 China

**Keywords:** Early gastric cancer, Lymph node metastasis, Prediction model, Meta-analysis, Systematic review

## Abstract

**Background:**

The number of risk prediction models for lymph node metastasis in early gastric cancer is increasing, but the quality and applicability of these models in clinical practice and future research remain unknown.

**Objective:**

To systematically review studies published on prediction models for the risk of lymph node metastasis in early gastric cancer patients.

**Design:**

Systematic review and meta-analysis of observational studies.

**Methods:**

A search was conducted in databases. Data from selected studies were extracted, including study design, data sources, outcome definitions, sample size, predictive factors, model development, and performance indicators. The risk of bias in prediction models was assessed using the Prediction model Risk Of Bias ASsessment Tool (PROBAST) checklist.

**Results:**

Fifty articles were included in this meta-analysis. Most studies used Logistic Regression (LR) to establish risk prediction models. The training model's overall c-statistic was 0.85 (95% CI (0.81–0.89)), whereas the validation model's overall c-statistic was 0.82 (95% CI (0.80–0.83)). The overall pooled accuracy rate for the training group model was 0.80 [95% CI (0.72–0.87)], and the overall accuracy rate for model validation was 0.71 [95% CI (0.61–0.79)]. Tumor size was the most common risk predictive factor. All studies had a high risk of bias, primarily due to inappropriate data sources.

**Conclusion:**

Based on the results of the PROBAST analysis, it was determined that all of the studies were highly biased. Models with bigger samples, more stringent research methods, inclusion of multicenter samples, and external validations should be the focus of future studies.

**Supplementary Information:**

The online version contains supplementary material available at 10.1186/s12876-025-04342-8.

## Background

Gastric cancer (GC) is the fifth most prevalent malignant tumor globally and is the third biggest contributor of cancer-related mortality worldwide [1]. In China, according to the 2022 China Cancer Incidence and Mortality Report, gastric cancer is the third leading cause of cancer-related deaths and is the fourth most incident malignant tumor in men and the third in women in terms of mortality [2]. Early gastric cancer (EGC) typically refers to infiltrative carcinomas confined to the mucosal or submucosal layers, regardless of their size or the presence of lymph node metastasis. Compared to gastric cancer, EGC has a more favorable prognosis, with a 5-year survival rate as high as 92.6% after endoscopic resection [3]. Data collected by the China Gastric Cancer Surgery Alliance from multiple hospitals across the country between 2014 and 2018 show an increase in the proportion of EGC among gastric cancer patients in China [4].

Lymph node metastasis serves as an independent prognostic factor for gastric cancer patients and is instrumental in predicting overall postoperative survival outcomes [5]. Early gastric cancer can be treated with endoscopy [6], and the implementation of endoscopic resection (ER) requires an assessment of the patient's likelihood of lymph node metastasis. This procedure is primarily used for primary tumor lesions with a low possibility of lymph node metastasis (LNM) [7]. Endoscopic submucosal dissection (ESD) and endoscopic mucosal resection (EMR) are procedures that, compared to traditional subtotal gastrectomy, can reduce patient suffering, better preserve gastric function, improve patient quality of life, and have similar postoperative survival rates [8]. Therefore, the status of lymph node metastasis is not only useful for assessing patient prognosis but also for evaluating the progression of the disease, selecting more appropriate surgical procedures, reducing patient suffering, and improving therapeutic outcomes. The number of risk prediction models for lymph node metastasis in early gastric cancer has increased in recent years to enhance the assessment of lymph node metastasis status. However, the quality and applicability of these models in clinical practice and future research remain unknown, necessitating systematic evaluation.

## Methods

### Search strategy

Our exhaustive search included databases such as PubMed, Embase, and the Cochrane Library, with a cutoff date of May 1, 2024. The search terms included "early gastric cancer," "lymph node metastasis," "risk prediction models," "risk factors," "prediction models," and "risk scores," with articles limited to Chinese and English languages. We supplemented the literature list by reviewing the reference lists of consulted articles and relevant review articles, thereby adding pertinent literature to the list.

We used the PICOTS system for systematic evaluation of the literature, which comes from the Critical Appraisal and Data Extraction for Systematic Reviews of Prediction Modelling Studies (CHARMS) [9]checklist. This system can be used to plan evaluation objectives, search strategies, and inclusion and exclusion criteria for literature. The main items evaluated in this paper are as follows:

- P (Population): People who are in the early stages of stomach cancer.
- I (Intervention Model): Completed development and published risk prediction models for lymph node metastasis in early gastric cancer patients (with at least 2 predictors).
- C (Comparison Model): No competing models.
- O (Outcome): The results focus on early gastric cancer, not its subgroups.
- T (Timing): Predicting outcomes after assessing basic information and laboratory indicators at the time of patient admission.
- S (Setting): Predicting lymph node metastases in early gastric cancer patients on an individual basis can help with treatment approach selection and prognosis evaluation.

### Inclusion and exclusion criteria

Inclusion criteria: (1) Patients diagnosed with early gastric cancer through histopathological examination; (2) Full-text articles available; (3) Clear reporting of the effectiveness of LNM prediction algorithms; (4) Sufficient data in the article to assess the c-statistic and/or accuracy.

Exclusion criteria: (1) Studies unrelated to EGC and/or LNM; (2) Animal studies, conference abstracts, consensus, case reports, and guidelines, etc.; (3) Instead than focusing on developing and validating prediction models, studies mostly sought to identify specific predictive elements; (4) Studies not reporting performance data for prediction models; (5) Single-variable prediction model performance; (6) Inability to obtain the full text after contacting the authors.

### Screening and selection of literature

Two reviewers, DM and WZS, conducted separate literature searches. Reviewing titles and abstracts for eligibility according to preset criteria followed the removal of duplicates. After going over the criteria for inclusion and exclusion, we read the full texts of the included works and looked through their reference lists to make sure we didn't miss anything. If there was a disagreement between the two authors on individual literature, a third author was added, and the three authors (DM, WZS, and X) discussed and determined together.

### Data extraction

Two reviewers (DM and WZS) independently screened the literature, and if there were differences, a third reviewer (X) joined, and the three discussed and resolved the issue. The data extraction checklist was developed in accordance with the modified prediction model study systematic review critical appraisal and data extraction checklist (CHARMS). The extracted data included: (1) Essential details of the literature: encompassing author, publication date, and geographical location; (2) Number of participants, the amount of positive lymph node metastasis tests, and kind of cancer are all parameters of the cohort.; (3) Variable selection algorithm, development method, variable processing method, number and type of final predictive factors, model validation type; (4) Performance indicators, including c-statistic, accuracy, sensitivity, and specificity.

### Quality assessment

Using two techniques, namely the Grading of Recommendations Assessment, Development and Evaluation (GRADE) [10] and the Prediction model Risk Of Bias Assessment Tool (PROBAST) [11], we were able to detect the quality and potential bias risk of the included research. The GRADE method is used to assess the quality of evidence and the degree of recommendation, which can classify the results of the study. The GRADE system categorizes evidence quality into four levels: high, moderate, low, and very low. It evaluates evidence quality based on six factors, which include study design, risk of bias, inconsistency, indirectness, imprecision, and additional considerations such as publication bias. The PROBAST checklist is mainly used to check the bias risk and applicability to the target population and environment of the study and can be used to assess prediction model studies. It is mainly divided into four parts, including participants, predictive factors, outcomes, and analysis, and obtains answers through 20 signal questions. The response to each signal question comprises "yes," "probably," "no," "probably not," and "no information." A section is deemed to have a high bias risk if at least one signal question is answered with "no" or "probably not." An overall low risk should manifest as all internal components being at low risk of bias.

The assessment process for each study was completed independently by two authors (DM and WZS), and if there was a disagreement, a third reviewer (X) would intervene, and they would discuss and resolve the issue together.

### Data synthesis and statistical analysis

Meta-analyses of model performance metrics, including c-statistic and accuracy, were conducted using R 4.2.2 and Stata 17.0 (StataCorp, TX, USA).

The I^2^ index and Cochrane Q test were used to assess heterogeneity. The I^2^ index was used as a numerical expression of heterogeneity, with 25%, 50%, and 75% representing low, moderate, and high heterogeneity [12], respectively. When the I^2^ index ≥ 50%, a random effects model was employed, otherwise, a fixed effects model was used. The Egger test was utilized to evaluate publication bias, with a p-value above 0.05 suggesting a reduced likelihood of publication bias [13]. The sensitivity analysis was conducted to evaluate the stability of the meta-analysis. All reported P values were two-sided with a statistical significance level of 0.05.

## Results

### Included literature

**Fig. 1:**
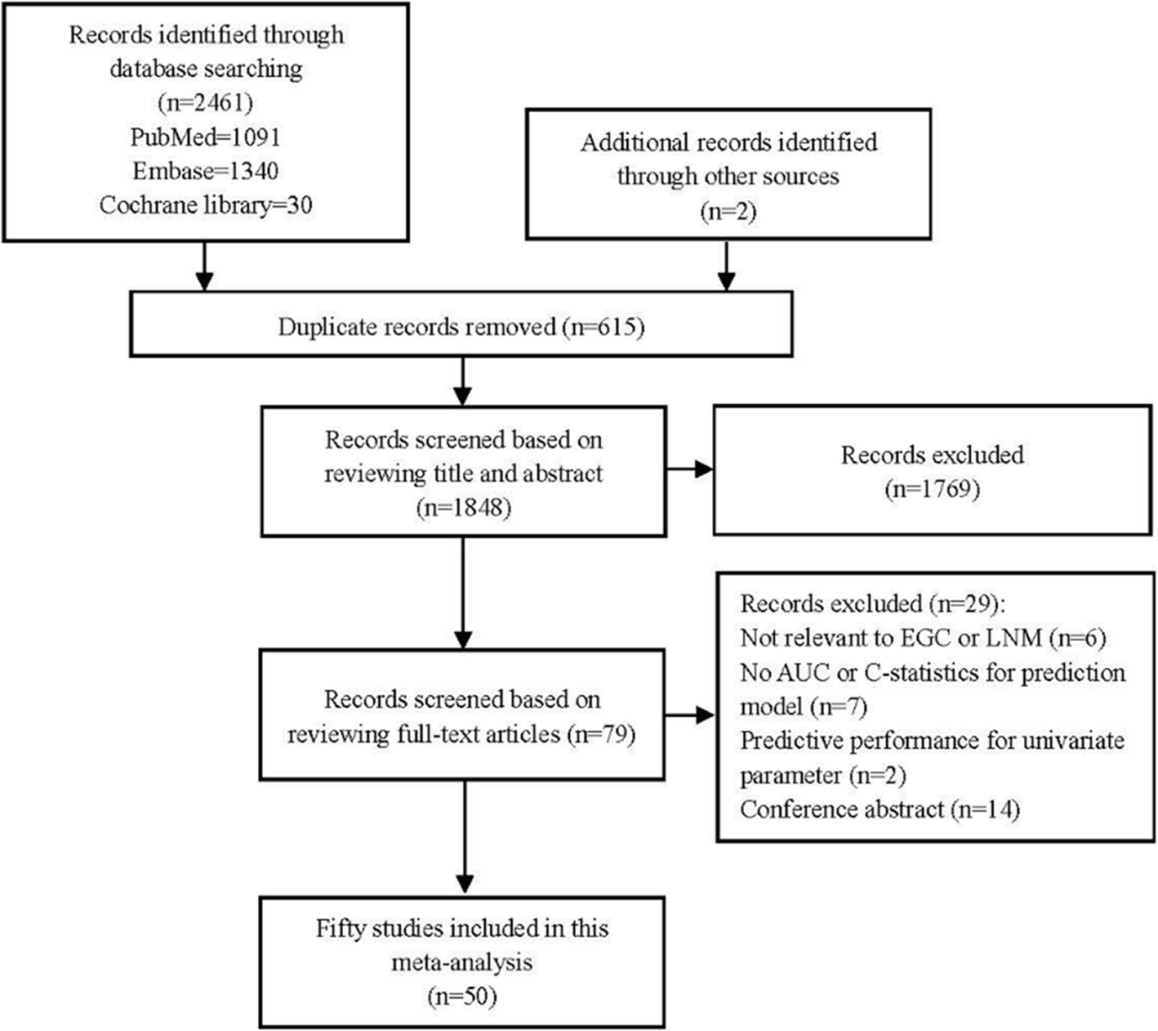
Flowchart of the selection of eligible studies

A search through three databases using relevant keywords yielded a total of 2461 articles, with 1091 from PubMed, 1340 from Embase, and 30 from the Cochrane Library, plus 2 additional articles from other sources. After checking for duplicates, 615 records were excluded. Further review of titles and abstracts led to the exclusion of 1769 articles. A comprehensive review of the remaining 79 articles was conducted, resulting in the exclusion of 29 articles that did not meet the established criteria, including 6 articles deemed unrelated to EGC or LNM.; 7 for lacking AUC or C-statistic measures; 2 for being single-variable prediction models; and 14 for being conference abstracts. Ultimately, 50 articles were incorporated in this meta-analysis (Fig. 1).

### Study characteristics

Table 1 presents the design and participant characteristics of the 50 studies that comprise this review. Studies were published from 2015 to 2024, which includes 42 conducted in China, 7 in South Korea, and 1 in Japan. Among the included studies, 49 were retrospective, including 20 multicenter studies; one was a combined retrospective and prospective study conducted in a single center. In terms of study subjects, there were 3 studies targeting signet ring cell carcinoma patients, and one study each targeting intestinal-type early gastric cancer, proximal early gastric cancer, poorly differentiated mucosal gastric cancer, submucosal invasive gastric cancer, early gastric signet ring cell carcinoma, and submucosal gastric cancer patients, sample sizes varied from 183 to 12,679 participants.

**Table 1:** Design characteristics of the included studies

No	First author	Year of publication	Country	Study design	No. of centres	Period of data collection	No. of patients		Cancer Type
							**Training set**	**Validation set**	
1	Wang	2024	China	Retro	Single	2016–2018	215	92	Intestinal-type EGC
2	Zhao	2023	China	Retro	Multiple	2011–2019	2215	943	EGC
3	You	2023	China	Retro	Single	2014.1–2022.6	183		Early GSRCC
4	Yang	2023	China	Retro	Single	2012.1–2022.3	1922		EGC
5	Wu	2023	China	Retro	Single	2016–2021	743	318	EGC
6	Li J	2023	China	Retro	Multiple	2004–2015	2797	1302	EGC
7	Li C	2023	China	Retro	Single	2014.1–2022.1	626		EGC
8	Lee	2023	Korea	Retro	Single	2012.1–2017.6	2044	1060	EGC
9	Jiang	2023	China	Retro	Multiple	2010–2015	1550	667	EGC
10	Dong	2023	China	Retro	Multiple	—	102	245	EGC
11	Zhu	2022	China	Retro	Multiple	—	1878		EGC
12	Zhang	2022	China	Retro	Single	2012.1–2021.6	952		EGC
13	Zeng	2022	China	Retro	Single	2016.8–2021.11	388	246	EGC
14	Yang	2022	China	Retro	Multiple	2012.1–2021.3	305	35	EGC
15	So	2022	Korea	Retro	Single	2003–2017	683	342	Proximal EGC
16	Na	2022	Korea	Retro	Single	2005.5–2021.3	10,332	4428	EGC
17	Liu	2022	China	Retro	Single	2017.7–2021.12	292		EGC
18	Li	2022	China	Retro	Multiple	2015–2020	505	831	EGC
19	Guo	2022	China	Retro	Single	2013–2020	226		EGC
20	Cui	2022	China	Retro	Multiple	2005.12–2020.12	1633	239	EGC
21	Chen	2022	China	Retro	Multiple	2015–2020	302	130	EGC
22	Cai	2022	China	Retro	Single	2016–2019	351	129	EGC
23	Zhou	2021	China	Retro	Multiple	—	818	351	Poorly differentiated‑type intramucosal GC
24	Zhang	2021	China	Retro	Single	2010–2015	285	—	EGC
25	Wei	2021	China	Retro	Single	2010.11–2019.12	548	73	EGC
26	Wang	2021	China	Retro	Single	2012.8–2019.8	363	140	EGC
27	Tian	2021	China	Retro	Multiple	2010–2016	2294	227	EGC
28	Sui	2021	China	Retro	Multiple	2011.11–2021.4	1496	246	EGC
29	Pan	2021	China	Retro	Multiple	1980–2012 2000–2016	1274	637	EGC
30	Mei	2021	China	Retro	Single	2012.1–2018.08	794	418	EGC
31	Jin	2021	China	Retro	Single	2017.1–2019.6	172	—	EGC
32	Gao	2021	China	Retro	Single	2012.5–2018.12	308	155	EGC
33	Yin	2020	China	Retro	Single	2015.1–2019.1	596	227	EGC
34	Kim	2020	Korea	Retro	Single	2001–2015	10,579	2100	EGC
35	Mu	2019	China	Retro	Single	2010.1–2018.7	746	126	EGC
36	Ma	2019	China	Retro	Multiple	2012–2018	120	281	submucosal-invasive GC
37	Lin	2019	China	Retro	Multiple	1990–2016	1460	172	EGC
38	Chen	2019	China	Retro	Multiple	2008–2013	232	143	EGC
39	Zhang	2018	China	Retro	Single	2010.11–2016.7	272	81	EGC
40	Gu	2018	China	Retro	Multiple	2000–2014	1029	394	EGC
41	Lou	2017	China	Retro + Pros	Single	2006–2015	312	89	EGC
42	Jung	2017	Korea	Retro	Multiple	2007.1–2015.1	321	321	EGC
43	Guo	2017	China	Retro	Single	2002–2014	952	—	EGC
44	Zheng	2016	China	Retro	Single	1996.12–2012.12	597	—	EGC
45	Zhao	2016	China	Retro	Single	2000–2013	598	—	EGC
46	Sekiguchi	2016	Japan	Retro	Single	1997.7–2013.5	3131	352	EGC
47	Pyo	2016	Korea	Retro	Multiple	2002–2013	772	980	Signet ring cell-type intramucosal GC
48	Guo	2016	China	Retro	Multiple	2002–2015 1946–2007	256	1273	Early GSRCC
49	Eom	2016	Korea	Retro	Single	2006.1–2006.9	336	—	EGC
50	Zheng	2015	China	Retro	Single	1996–2012	262	—	Submucosal GC

*EGC* Early gastric cancer, *GC* Gastric cancer, *GSRCC* Gastric signet ring cell carcinoma, *Retro* Retrospective, *Pros* Prospective

Table 2 presents the performance characteristics of the prediction models. According to the table, most studies utilized Logistic Regression (LR) to establish prediction models. Additionally, Zhao et al. [15] employed Nomogram, Decision Tree (DT), Naive Bayes, and Neural Network (NNET) methods; Zeng et al. [24] utilized Support Vector Machine (SVM), K-Nearest Neighbor (KNN), Random Forest (RF), and Extreme Gradient Boosting (XGBoost) techniques; Tian et al. [35] modeled utilizing Generalized Linear Model (GLM), Recursive Partitioning and Regression Trees (RPART), Random Forest (RF), Gradient Boosting Machine (GBM), SVM, Ridge Discriminant Analysis (RDA), andNNET methods. Zhu et al. [23] used LR and incorporated GBM, XGBoost, RF, Decision Tree (DT), and NNET methods in their analysis.

**Table 2:** Study and performance characteristics of the prediction models

No	Study	Method for variable selection	Model method	C-statistics/AUC		Accurary	Calibration	Model validation	No. of predictors
				**Training set**	**Validation set**				
1	Wang 2024 [14]	LR	LR	0.872 (0.722**–**0.909)	0.885 (0.682**–**1.0)	—	—	External	5
2	Zhao 2023 [15]	LR	Nomogram, DT, Naive bayes, NNET	Nomogram: 0.8 Decision tree: 0.79	Nomogram: 0.78 Decision tree: 0.76 Naive bayes: 0.77 FCNN: 0.79	—	Calibration curve	Internal	6
3	You 2023 [16]	Lasso + LR	LR	—	0.757 (0.687**–**0.828)		Calibration curve	Internal	4
4	Yang 2023 [17]	LR, NNET	LR, NNET	LR: 0.797 (0.776**–**0.818) NNET: 0.878 (0.862**–**0.893)	LR: 0.816 (0.794**–**0.839) NNET: 0.899	Training: LR: 0.867 (0.851**–**0.882) NNET: 0.882 (0.868**–**0.898)	Calibration curve	Internal	6
5	Wu 2023 [18]	Lasso + LR	LR	0.775 (0.734**–**0.816)	0.792 (0.729**–**0.855)	—	Hosmer–Lemeshow; Calibration curve	Internal	4
6	Li J. 2023 [19]	LR	LR	0.702 (0.679**–**0.725)	Internal: 0.709 (0.674**–**0.744) External: 0.750 (0.607**–**0.892)	—	—	Internal/External	4
7	Li C. 2023 [20]	LR	LR	0.899 (0.724**–**0.915)	—	—	Hosmer–Lemeshow	—	4
8	Lee 2023 [21]	LR	LR, GBM	—	Internal: LR: 0.876 (0.827**–**0.918) GBM: 0.867 (0.812**–**0.918) External: LR: 0.943 (0.909**–**0.971) GBM: 0.944 (0.914–0.97)	Internal: LR: 0.617 (0.593**–**0.629) GBM: 0.566 (0.542**–**0.579) External: LR: 0.803 (0.787**–**0.806) GBM: 0.81 (0.794**–**0.814)	—	Internal/External	9
9	Jiang 2023 [19	LR	LR	0.751 (0.721–0.782)	0.786 (0.742–0.830)	—	Calibration curve	Internal	5
10	Dong 2023 [22]	LR	LR	0.961	TCGA: 0.852 ACRG: 0.834	—	—	—	10
11	Zhu 2022 [23]	—	LR, GBM, XGBoost, RF, DT, NNET	GBM: 0.791 XGBOOST: 0.788 LR: 0.783 NNET: 0.768 RF: 0.749 DT: 0.733	—	—	—	—	7
12	Zhang 2022	Lasso + LR	LR	0.816 (0.781–0.853)	0.805 (0.770–0.842)	—	Calibration curve	Internal	8
13	Zeng 2022 [24]	Lasso	SVM, KNN, RF, XGBoost	SVM: 0.909 (0.874**–**0.943) KNN: 0.885 (0.854**–**0.916) RF: 0.999 (0.998**–**1) XGBoost: 0.999 (0.999**–**1)	Internal: SVM: 0.901 (0.847**–**0.956) KNN: 0.793 (0.713**–**0.874) RF: 0.811 (0.742**–**0.88) XGBoost: 0.821 (0.742**–**0.9) External: SVM: 0.916 (0.85**–**0.981) KNN: 0.878 (0.792**–**0.964) RF: 0.72 (0.611**–**0.83) XGBoost: 0.84 (0.716**–**0.964)	SVM: 0.83 KNN: 0.812 RF: 0.989 XGBoost: 0.987	—	Internal/External	9
14	Yang 2022 [25]	Lasso LR	SVC, LR, XGBoost, GBM, Gaussian Process	SVC: 0.786 (0.783**–**0.789) LR: 0.788 (0.785**–**0.79) XGBoost: 0.781 (0.778**–**0.784) GBM: 0.766 (0.763**–**0.769) GP: 0.816 (0.813**–**0.819)	SVC: 0.736 (0.731**–**0.741) LR: 0.732 (0.727**–**0.738) XGBoost: 0.804 (0.799**–**0.809) GBM: 0.83 (0.826**–**0.835) GP: 0.803 (0.799**–**0.808)	Training: SVC: 0.723 (0.720**–**0.725) LR: 0.806 (0.804**–**0.808) XGBoost: 0.796 (0.794**–**0.798) GBM: 0.714 (0.712**–**0.717) GP: 0.815 (0.813–0.817) External: SVC: 0.486 (0.482**–**0.489) LR: 0.798 (0.795**–**0.801) XGBoost: 0.826 (0.823**–**0.828) GBM: 0.566 (0.563**–**0.569) GP: 0.771 (0.768**–**0.774)	—	External	7
15	So 2022 [54	LR	LR	0.85	0.84	—	—	Internal	4
16	Na 2022 [1]	—	LR, RF, SVM	LR: 0.86 (0.85**–**0.88) RF: 0.95 (0.94**–**0.95) SVM: 0.87 (0.85**–**0.88)	LR: 0.86 (0.84**–**0.88) RF: 0.85 (0.83**–**0.87) SVM: 0.86 (0.84**–**0.88)	—	—	Internal	12
17	Liu 2022	LR	LR	0.86	0.84	0.695	Hosmer–Lemeshow; Calibration curve	Internal	5
18	Li 2022 [26]	LR	LR	0.731 (0.682–0.779)	Internal: 0.766 (0.709**–**0.823) External: 0.625 (0.573**–**0.678)	Training: 0.588 (0.544**–**0.631) Internal: 0.588 (0.533**–**0.641) External: 0.617 (0.573**–**0.660)	—	Internal/External	3
19	Guo 2022 [27]	LR	LR	0.703 (0.633**–**0.773)	—	—	Calibration curve	—	3
20	Gui 2022	LR	LR	0.818 (0.790**–**0.847)	0.765 (0.688**–**0.843)	—	Calibration curve	External	7
21	Shen 2022	LR	LR	0.91 (0.87**–**0.95)	0.89 (0.81**–**0.96)	Training: 0.834 External: 0.862	—	External	5
22	Cai 2022 [28]	LR	LR	0.839 (0.769**–**0.91)	0.82 (0.711**–**0.93)	—	—	External	4
23	Zhou 2021 [29]	GBDT	RF, LR, XGB, GBDT	RF: 0.802 GBDT: 0.798 XGB: 0.881 LR: 0.778	RF: 0.765 GBDT: 0.788 XGB: 0.762 LR: 0.75	Training: RF: 0.947 GBDT: 0.955 XGB: 0.949 LR: 0.946 External: RF: 0.949 GBDT: 0.946 XGB: 0.952 LR: 0.946	—	Internal	3
24	Zhang 2021 [30]	LR	LR	0.842 (0.795–0.882)	—	—	Hosmer–Lemeshow; Calibration curve	—	6
25	Wei 2021 [31]	LR	LR	0.834 (0.783**–**0.886)	0.829 (0.699**–**0.959)	—	Hosmer–Lemeshow	Internal	5
26	Wang 2021 [32]	LR	LR	—	0.803 (0.773**–**0.879)	—	Calibration curve	Internal	6
27	Tian 2021	LR	GLM, RPART, RF, GBM, SVM, RDA, NNET	GLM: 0.743 RPART: 0.721 RF: 0.722 GBM: 0.731 SVM: 0.7 RDA: 0.742 NNET: 0.758	RDA: 0.73	—	—	External	3
28	Sui 2021 [33]	LR	LR	Female: 0.877 (0.84**–**0.914) Male: 0.948 (0.927**–**0.97)	Female: 0.924 (0.798**–**1) Male: 0.934 (0.893**–**0.976)	—	Calibration curve	External	4
29	Pan 2021 [34]	LR	LR	0.723 (0.629**–**0.755)	0.706 (0.658**–**0.755)	—	Calibration curve	Internal	3
30	Mei 2021 [35]	LR	LR	0.76 (0.719**–**0.8)	0.771 (0.714**–**0.828)	—	Calibration curve	Internal	5
31	Jin 2021 [36]	LR	LR	0.886 (0.819**–**0.948)	—	—	Calibration curve	—	3
32	Gao 2021	LR	LR	0.87 (0.81**–**0.92)	0.85 (0.77–0.91)	—	Hosmer–Lemeshow; Calibration curve	Internal	2
33	Yin 2020 [37]	LR	LR	0.82 (0.78**–**0.86)	0.77 (0.6**–**0.94)	—	Calibration curve	External	6
34	Kim 2020 [38]	LR	LR	0.846 (0.833**–**0.859)	0.813 (0.786**–**0.84)	—	Calibration curve	External	5
35	Mu 2019	LR	LR	0.864 (0.827**–**0.901)	Internal: 0.861 (0.851**–**0.864) External: 0.911 (0.848**–**0.974)	—	Calibration curve	Internal/External	4
36	Ma 2019 [39]	Lasso CR	LR	0.872 (0.823**–**0.918)	Internal: 0.898 (0.866‐0.959) External: 0.829 (0.753‐0.907) 0.792 (0.731‐0.873)	—	—	Internal/External	4
37	Lin 2019 [40]	LR	LR	0.694 (0.659**–**0.73)	0.796 (0.662**–**0.851)	—	—	External	4
38	Chen 2019 [41]	Lasso + LR	LR	0.955 (0.919**–**0.991)	0.938 (0.897**–**0.981)	Training: 0.922 (0.899**–**0.961) External: 0.902 (0.843**–**0.955)	Hosmer–Lemeshow; Calibration curve	External	3
39	Zhang 2018 [42]	LR	LR	0.905	0.905	—	Calibration curve	External	4
40	Gu 2018 [43]	LR	LR	0.76 (0.73**–**0.8)	Internal: 0.77 (0.68**–**0.86) External: 0.82 (0.72**–**0.91) 0.82 (0.70**–**0.94)	—	—	Internal/External	6
41	Lou 2017 [44]	LR	LR	0.781 (0.721**–**0.841)	0.817 (0.714**–**0.92)	—	—	External	5
42	Jung 2017	LR	LR	0.811 (0.734**–**0.888)	Internal: 0.788 (0.662**–**0.914) External: 0.842 (0.753**–**0.932)	—	—	Internal/External	3
43	Guo 2017 [45]	LR	LR	0.786 (0.749**–**0.822)	—	—	Hosmer–Lemeshow; Calibration curve	—	5
44	Zheng 2016 [46]	LR	LR	0.86 (0.809**–**0.912)	—	—	—	—	8
45	Zhao 2016 [47]	LR	LR	0.847 (0.789**–**0.923)	—	—	Calibration curve	—	6
46	Sekiguchi 2016 [48]	LR	LR	0.84 (0.82**–**0.86)	0.82 (0.75**–**0.88)	—	—	External	5
47	Pyo 2016 [49]	LR	LR	0.70 (0.63**–**0.77)	Internal: 0.68 (0.58**–**0.79) External: 0.69 (0.62**–**0.75)	—	—	Internal/External	3
48	Guo 2016 [50]	LR	LR	0.801 (0.729**–**0.873)	0.707 (0.657**–**0.758)	—	Calibration curve	External	3
49	Eom 2016 [51]	LR	LR	0.865 (0.804**–**0.926)	—	—	—	—	4
50	Zheng 2015 [52]	LR	LR	0.844 (0.785**–**0.904)	—	—	Calibration curve	—	4

*AUC* Area under the curve, *LR* Logistic regression, *CR* Cox regression, *GBM* Gradient boosting machine, *NNET* Neural network, *DT* Decision tree, *RF* Random forest, *XGBOOST* Extreme gradient boosting, *GP* Gaussian process, *SVM* Support vector machine, *KNN* K‐nearest neighbor, *GLM* Generalized linear model

The AUC or C-statistic values reported in the studies varied from 0.694 to 0.999, with variations in AUC values explained by the methods utilized. Among these, 27 models indicated their calibration status, with the Calibration Curve test as the predominant method applied. The following was followed by studies making use of the Calibration Curve test in conjunction with the Hosmer–Lemeshow test, and those employing the Hosmer–Lemeshow test autonomously.

### Model validation

In the included studies, the majority of models underwent internal or external validation. Specifically, 15 studies conducted external validation, 15 conducted internal validation, 9 models included both internal and external validation, and 11 models did not undergo any validation.

### Bias risk and applicability assessment

Table 3 evaluates the risk of bias and the applicability of all studies included. All studies listed in the table have been classified as having a high risk of bias or an unclear risk, indicating methodological deficiencies in constructing or validation of the prediction models. Within the participant domain, 9 studies [1–9] (6、9、10、18、23、27、29、36、41) (18%) were considered to have a high risk of bias, primarily due to inappropriate data sources. In the predictor domain, all studies were at low risk of bias, demonstrating that the definition and measurement of predictive factors were genuinely reliable and similar across studies. In the outcome domain, all studies were assessed as low risk, indicating that the definition, measurement method, process, and timing of predictive outcomes were appropriate. In the analysis domain, 11 studies have been categorized as high risk. This involves 7 studies [3, 10–13, 15, 24] (10、11、19、44、45、49、50) that failed to comprehensively evaluate the predictive performance of their models and 4 studies [1, 20, 23, 35] (7、24、31、43) failed to offer details with respect to the coefficients of predictors in the multivariate regression model.

**Table 3:** Summary of risk of bias assessments for included studies based on the prediction model risk of bias assessment tool

No	Study	Study type	Risk of bias				Applicability			Overall	
			**Participants**	**Predictors**	**Outcome**	**Analysis**	**Participants**	**Predictors**	**Outcome**	**ROB**	**Applicability**
1	Wang 2024 [14]	A	−	+	+	?	+	+	+	−	+
2	Zhao 2023 [15]	A	−	+	+	+	+	+	+	−	+
3	You 2023 [16]	A	−	+	+	+	+	+	+	−	+
4	Yang 2023 [17]	A	−	+	+	+	+	+	+	−	+
5	Wu 2023 [18]	A	−	+	+	+	+	+	+	−	+
6	Li J. 2023 [19]	A	+	+	+	?	+	+	+	?	+
7	Li C. 2023 [20]	B	−	+	+	−	+	+	+	−	+
8	Lee 2023 [21]	A	−	+	+	?	+	+	+	−	+
9	Jiang 2023 [53]	A	+	+	+	+	+	+	+	+	+
10	Dong 2023 [22]	B	+	+	+	−	+	−	+	−	−
11	Zhu 2022 [23]	B	−	+	+	−	+	+	+	−	+
12	Zhang 2022	A	−	+	+	+	+	+	+	−	+
13	Zeng 2022 [24]	A	−	+	+	?	+	+	+	−	+
14	Yang 2022 [25]	A	−	+	+	?	+	+	+	−	+
15	So 2022 [54]	A	−	+	+	?	+	+	+	−	+
16	Na 2022 [1]	A	−	+	+	?	+	+	+	−	+
17	Liu 2022	A	−	+	+	+	+	+	+	−	+
18	Li 2022 [26]	A	+	+	+	?	+	+	+	?	+
19	Guo 2022 [27]	B	−	+	+	−	+	+	+	−	+
20	Gui 2022	A	−	+	+	+	+	+	+	−	+
21	Shen 2022	A	−	+	+	?	+	+	+	−	+
22	Cai 2022 [28]	A	−	+	+	?	+	+	+	−	+
23	Zhou 2021 [29]	A	+	+	+	?	+	+	+	?	+
24	Zhang 2021 [30]	B	−	+	+	−	+	+	+	−	+
25	Wei 2021 [31]	A	−	+	+	+	+	+	+	−	+
26	Wang 2021 [32]	A	−	+	+	+	+	+	+	−	+
27	Tian 2021	A	+	+	+	?	+	+	+	?	+
28	Sui 2021 [33]	A	−	+	+	+	+	+	+	−	+
29	Pan 2021 [34]	A	+	+	+	+	+	+	+	+	+
30	Mei 2021 [35]	A	−	+	+	+	+	+	+	−	+
31	Jin 2021 [36]	B	−	+	+	−	+	+	+	−	+
32	Gao 2021	A	−	+	+	+	+	+	+	−	+
33	Yin 2020 [37]	A	−	+	+	+	+	+	+	−	+
34	Kim 2020 [38]	A	−	+	+	+	+	+	+	−	+
35	Mu 2019	A	−	+	+	+	+	+	+	−	+
36	Ma 2019 [39]	A	+	+	+	?	+	−	+	?	−
37	Lin 2019 [40]	A	−	+	+	?	+	+	+	−	+
38	Chen 2019 [41]	A	−	+	+	+	+	+	+	−	+
39	Zhang 2018 [42]	A	−	+	+	+	+	+	+	−	+
40	Gu 2018 [43]	A	−	+	+	?	+	+	+	−	+
41	Lou 2017 [44]	A	+	+	+	?	+	+	+	?	+
42	Jung 2017	A	−	+	+	?	+	+	+	−	+
43	Guo 2017 [45]	B	−	+	+	−	+	+	+	−	+
44	Zheng 2016 [46]	B	−	+	+	−	+	+	+	−	+
45	Zhao 2016 [47]	B	−	+	+	−	+	+	+	−	+
46	Sekiguchi 2016 [48]	A	−	+	+	?	+	+	+	−	+
47	Pyo 2016 [49]	A	−	+	+	?	+	+	+	−	+
48	Guo 2016 [50]	A	−	+	+	+	+	+	+	−	+
49	Eom 2016 [51]	B	−	+	+	−	+	+	+	−	+
50	Zheng 2015 [52]	B	−	+	+	−	+	+	+	−	+

A indicates “development only”; B indicates “development and validation in the same publication”

*ROB* Risk of bias

+ Indicates low ROB/low concern regarding applicability, − Indicates high ROB/high concern regarding applicability, ? Indicates unclear ROB/unclear concern regarding applicability

In terms of applicability risk assessment, 2 studies [3, 8] (10,、36) were assessed as high risk, and 48 studies were assessed as low risk. The main reason for the 2 studies assessed as high risk in the predictor domain was issues with the setting of predictive factors. Two studies had a higher risk in "overall applicability," as shown in Table 3.

### Risk factors

**Fig. 2:**
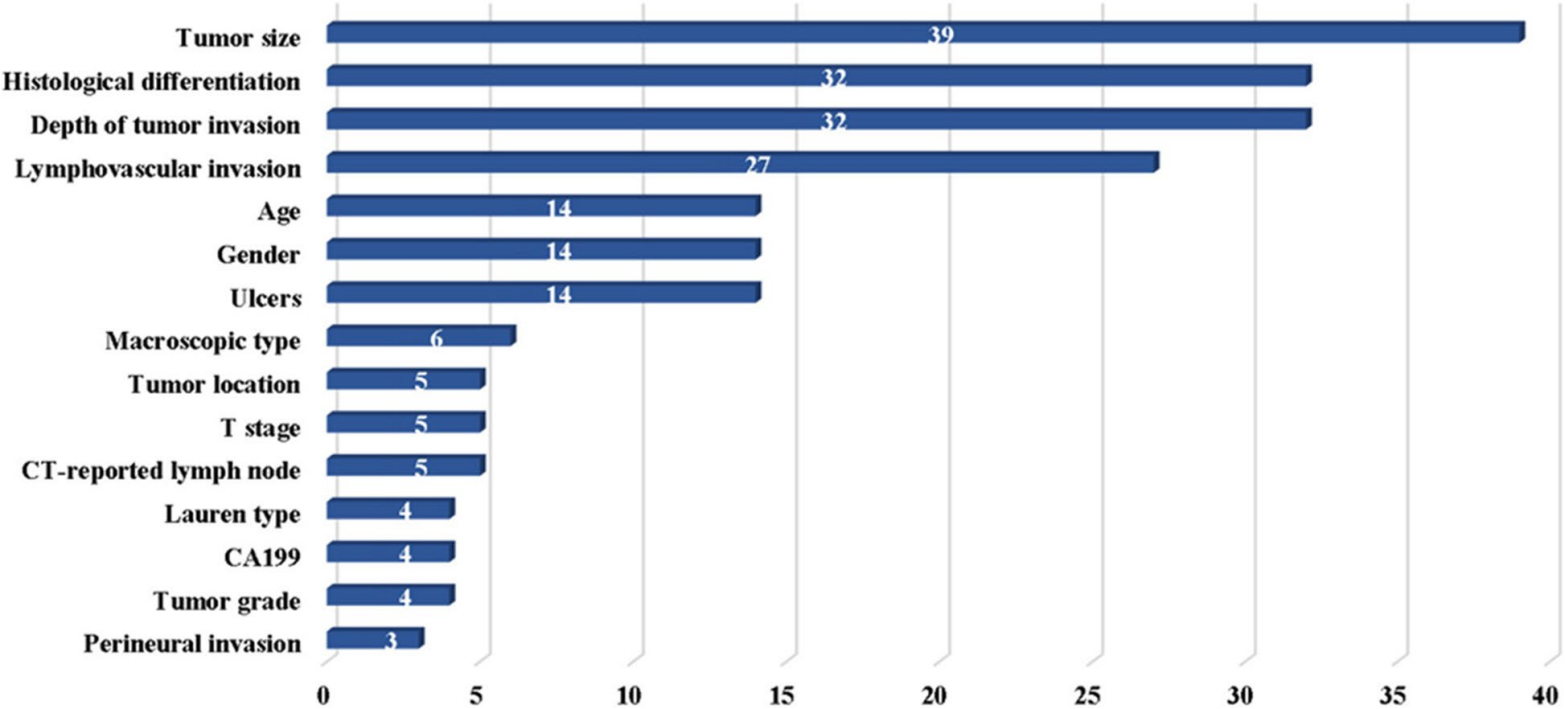
Risk factors of EGC included in the models

The risk factors included in the models encompass tumor size, tissue differentiation, tumor invasion depth, lymphatic invasion, age, gender, presence of ulcers, tumor typing, location, T stage, CT-reported lymph node metastasis, Lauren type, CA199, grading, and perineural invasion, among others. Tumor size was the most common risk predictor (Fig. 2).

### Predictive performance

Table 4 presents the C- statistics of models derived from multiple studies. The overall C-statistic for the training models was 0.85 [95% CI (0.81–0.89)] and there was significant heterogeneity among these included models (*P* < 0.001; *I*^2^ = 99.9%). The pooled C-statistic for Logistic Regression (LR) models was 0.82 [95% CI (0.80–0.84)], whereas the pooled C-statistic for non-Logistic Regression (non-LR) models was 0.93 [95% CI (0.78–0.98)]. The overall C-statistic for model validation was 0.82 [95% CI (0.80–0.83)] and significant heterogeneity was found among these included models (*P* < 0.001; *I*^2^ = 97.4%). The pooled C-statistic for internal validation was 0.81 [95% CI (0.79–0.83)], while the pooled C-statistic for external validation was 0.82 [95% CI (0.79–0.85)].

**Table 4:** C-statistics of models for predicting lymph node metastasis in patients with early gastric cancer

Model	No. of study	No. of model	C-statistics (95%CI)	C-statistics (95%PI)
Training set	38	49	0.85 (0.81–0.89)	0.85 (0.52–0.97)
Models				
LR	37	38	0.82 (0.80–0.84)	0.82 (0.67–0.92)
Non-LR	4	11	0.93 (0.78–0.98)	0.93 (0.12–1.00)
Country				
China	32	41	0.86 (0.81–0.89)	0.86 (0.47–0.98)
Other countries	6	8	0.86 (0.79–0.91)	0.86 (0.57–0.96)
Centres				
Single	24	30	0.88 (0.82–0.92)	0.88 (0.45–0.99)
Multiple	14	19	0.81 (0.76–0.85)	0.81 (0.58–0.93)
Validation				
Non-validated	9	9	0.83 (0.79–0.87)	0.83 (0.68–0.92)
Internally validated	9	12	0.84 (0.78–0.88)	0.84 (0.59–0.95)
Externally validated	13	18	0.83 (0.79–0.86)	0.83 (0.65–0.93)
Internally and externally validated	7	10	0.93 (0.72–0.98)	0.93 (0.06–1.00)
Validation set	33	59	0.82 (0.80–0.83)	0.82 (0.65–0.91)
Models				
LR	32	43	0.81 (0.78–0.83)	0.81 (0.64–0.91)
Non-LR	4	16	0.84 (0.80–0.87)	0.84 (0.68–0.92)
Internal validation				
All models	20	26	0.81 (0.79–0.83)	0.81 (0.69–0.89)
LR	19	19	0.80 (0.77–0.83)	0.80 (0.66–0.89)
Non-LR	2	7	0.85 (0.83–0.87)	0.85 (0.83–0.87)
External validation				
All models	22	33	0.82 (0.79–0.85)	0.82 (0.61–0.93)
LR	21	24	0.82 (0.77–0.85)	0.82 (0.59–0.93)
Non-LR	3	9	0.83 (0.76–0.89)	0.83 (0.56–0.95)
Country				
China	27	46	0.80 (0.78–0.82)	0.80 (0.67–0.89)
Other countries	6	13	0.85 (0.79–0.89)	0.85 (0.61–0.95)
Centres				
Single	18	31	0.84 (0.82–0.86)	0.84 (0.73–0.91)
Multiple	15	28	0.78 (0.75–0.81)	0.84 (0.73–0.91)

*CI* Confidence interval, *PI* Prediction interval, *LR* Logistic regression

Table 5 summarizes the accuracy of the included models, stratified by training and validation sets, with further subgroup analyses by model type (logistic regression [LR] vs. non-LR) and study design (single-center vs. multi-center), along with their 95% confidence intervals (95%CI) and 95% prediction intervals (95%PI).

**Table 5:** Accuracy of models for predicting lymph node metastasis in patients with early gastric cancer

Model	No. of study	No. of model	Accurary (95%CI)	Accurary (95%PI)
Training set	4	9	0.80 (0.72–0.87)	0.80 (0.47–0.95)
Models				
LR	4	4	0.82 (0.53–0.95)	0.82 (0.06–1.00)
Non-LR	2	5	0.79 (0.69–0.87)	0.79 (0.45–0.95)
Centres				
Single	1	2	0.87 (0.74–0.94)	0.87 (NaN- NaN)
Multiple	3	7	0.78 (0.66–0.86)	0.78 (0.40–0.95)
Validation set	4	12	0.71 (0.61–0.79)	0.71 (0.34–0.92)
Models				
LR	4	6	0.73 (0.57–0.85)	0.73 (0.27–0.95)
Non-LR	2	6	0.69 (0.51–0.82)	0.69 (0.21–0.95)
Centres				
Single	1	4	0.71 (0.48–0.87)	0.71 (0.11–0.98)
Multiple	3	8	0.71 (0.56–0.82)	0.71 (0.26–0.94)

*CI* Confidence interval, *PI* Prediction interval, *LR* Logistic regression

In the training set (4 studies, 9 models), the pooled accuracy was 0.80 (95%CI 0.72–0.87; 95%PI 0.47–0.95). By model type, LR models (4 studies, 4 models) had an accuracy of 0.82 (95%CI 0.53–0.95; 95%PI 0.06–1.00), slightly higher than non-LR models (2 studies, 5 models; 0.79, 95%CI 0.69–0.87; 95%PI 0.45–0.95). By study design, single-center models (1 study, 2 models) showed a higher accuracy of 0.87 (95%CI 0.74–0.94; 95%PI NaN-NaN) compared to multi-center models (3 studies, 7 models; 0.78, 95%CI 0.66–0.86; 95%PI 0.40–0.95).

In the validation set (4 studies, 12 models), the overall accuracy decreased to 0.71 (95%CI 0.61–0.79; 95%PI 0.34–0.92). LR models (4 studies, 6 models) maintained a marginal advantage over non-LR models (2 studies, 6 models), with accuracies of 0.73 (95%CI 0.57–0.85; 95%PI 0.27–0.95) and 0.69 (95%CI 0.51–0.82; 95%PI 0.21–0.95, respectively). Notably, single-center models (1 study, 4 models) and multi-center models (3 studies, 8 models) in the validation set achieved comparable accuracies (both 0.71), with 95%CI of 0.48–0.87 and 0.56–0.82, respectively.

**Fig. 3:**
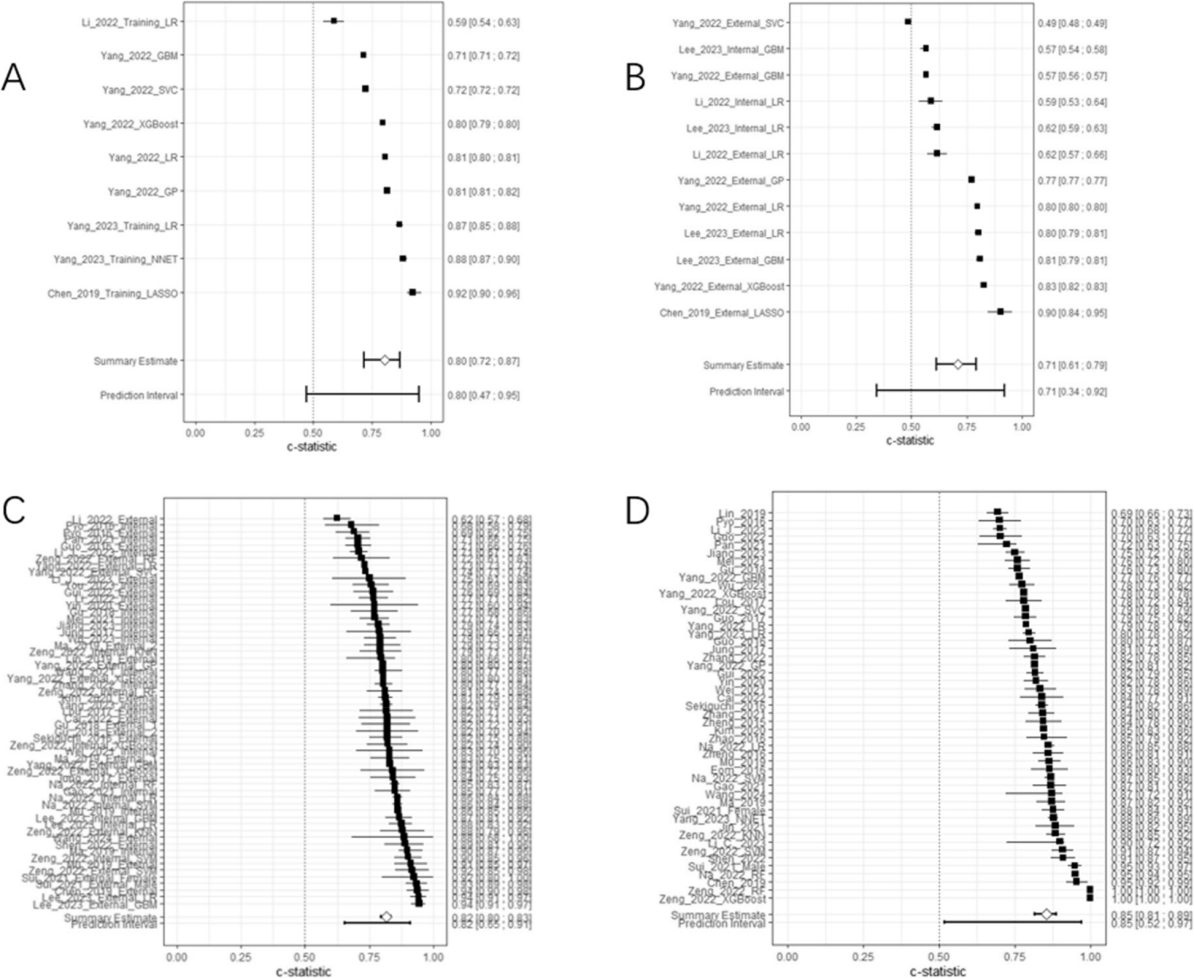
**A** Forest plot of accuracy for the training set; **B** Forest plot of accuracy for the validation set; **C** Forest plot of the validation set; **D** Forest plot of the training set

Overall, models performed better in the training set than in the validation set. LR models consistently outperformed non-LR models in both sets, while single-center models showed higher accuracy in the training set but no advantage over multi-center models in the validation set. The wide ranges of 95%PI, particularly for LR models in the training set, indicate substantial variability in model performance (Fig. 3).

### Publication bias and sensitivity analysis

The publication bias for training models and validation models was evaluated by the Egger test. The results displayed that there was a high likelihood of publication bias for training models (P = 0.002) and a low evidence of publication bias for validation models (P = 0.320).

**Fig. 4:**
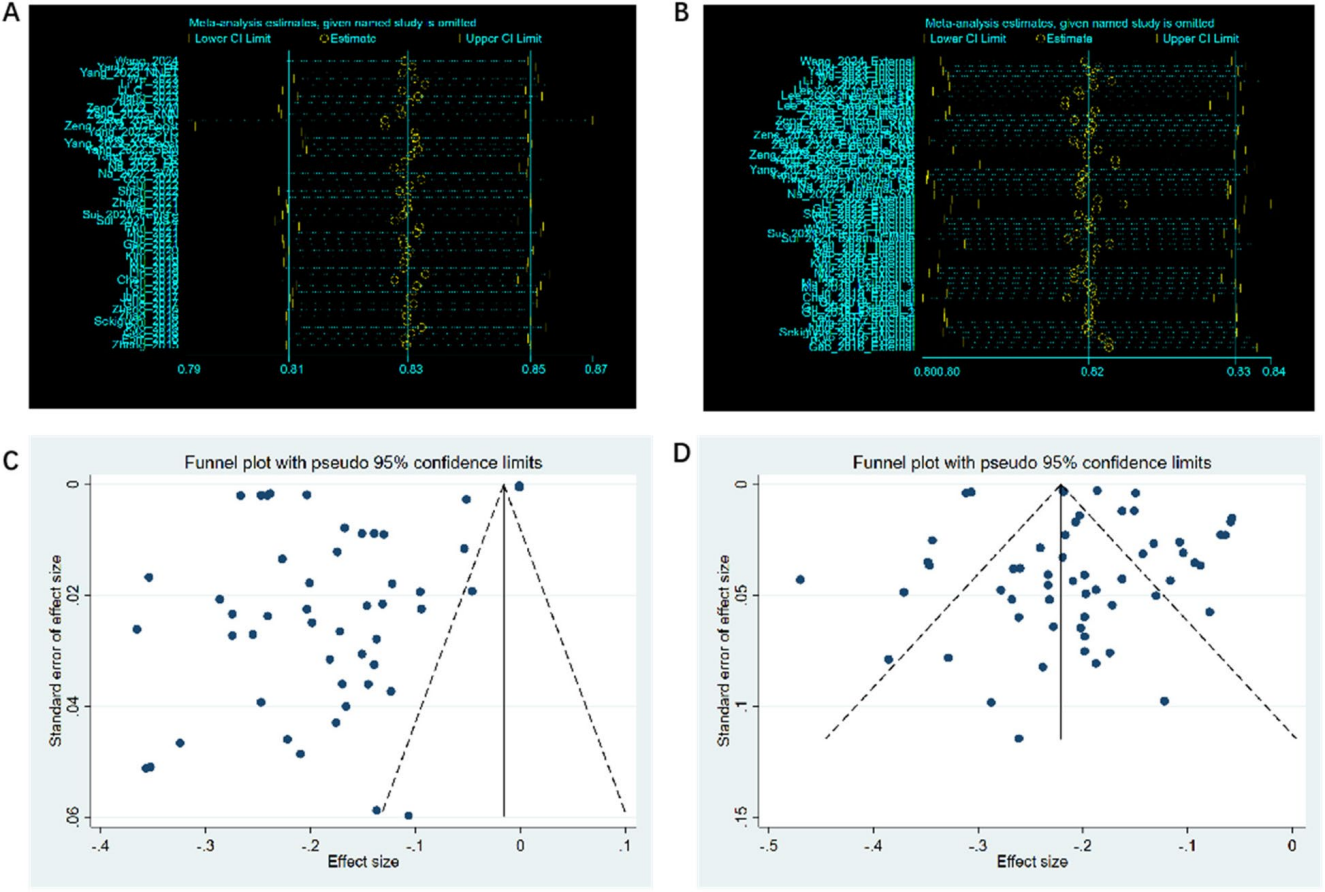
**A** Sensitivity analysis plot of training set; **B** Sensitivity analysis plot of validation set; **C** Funnel plot of training set; **D** Funnel plot of validation set

The results of the sensitivity analysis displayed that eliminating each individual study did not significantly influence the overall summary estimates, indicating that the results of the present meta-analysis were stable (Fig. 4).

## Discussion

According to estimates by the International Agency for Research on Cancer (IARC), about one in five individuals globally will develop cancer, and approximately one in nine men and one in twelve women will die from cancer, with gastric cancer ranking fifth in terms of incidence and mortality, and the number of affected individuals continues to rise rapidly [55]. Advanced gastric cancer is typically treated with subtotal gastrectomy, while early gastric cancer (EGC), due to its limited invasion, can be treated with endoscopic procedures [56]. Endoscopic treatment can better preserve gastric function and improve patients' quality of life, but its limited surgical scope may affect patients' surviva [57]. The status of lymph node metastasis can determine the prognosis of EGC [58],hence the preoperative assessment of lymph node metastasis and the guidance on the extent of lymph node dissection in the textbook are of extremely high clinical value for the endoscopic treatment of EGC.

This review evaluated 50 EGC lymph node metastasis risk models, the majority of which were based on data from Chinese patients. According to the retrieved data, the number of studies related to EGC lymph node metastasis risk models is increasing. In the 50 studies evaluated, the majority of models were validated post-establishment and exhibited moderate to good predictive performance in both internal and external validation, with AUC values reported between 0.70 and 0.999. All studies incorporated into the analysis have been evaluated using the PROBAST checklist and were judged to possess either a high risk of bias or an unclear risk, which limitations the practical applicability of these predictive models. The participant domain was judged to be at high risk of bias due to the use of inappropriate data sources. For example, some studies rely on specific regional registries or single-center retrospective data. Such data only reflect patient characteristics under specific regional and diagnostic/treatment patterns, and thus cannot well represent populations with different medical resources, ethnic groups, or disease courses. The significant heterogeneity among the models presented in this paper may stem from variations in the populations, regions, predictive factors, and methodologies employed across different models.

Beyond the predictive models themselves, we can also learn from the process of model establishment. For instance, Zhu et al. [23] developed models using Logistic Regression and Neural Network methods. Machine Learning (ML) algorithms, a branch of computer science, can process data more efficiently and accurately, analyzing connections between data to make more precise analytical predictions [59]. Machine learning is well-suited for constructing predictive models [60], compared to traditional logistic regression modeling, it is simpler and more verifiable, helping traditional research address issues such as the handling of continuous variables and the setting of predictive factors. However, applying machine learning requires a higher level of computer skills, and there can be significant differences between different algorithms, so researchers must carefully choose and complement each other to select the appropriate model development method [61]. In terms of data inclusion, most studies used data from a single center, leading to a limited training set and difficulty in generating broader public health value. Model researchers should consider using multicenter data to increase sample size and improve applicability. Although the models demonstrated moderate to good performance, they exhibited notable bias risks. This underscores the necessity for researchers to enhance their data source selection, predictive factor assessment, continuous variable management, data complexity handling, and model calibration practices.

This study's models demonstrate specific clinical values. Various models indicate that general factors, including age and gender, may act as risk factors for lymph node metastasis in early gastric cancer (EGC); however, the conclusions drawn from these models are inconsistent. Most studies believe that female and younger patients have a higher risk of lymph node metastasis [17, 19, 23, 40, 43, 50, 53], which is consistent with some clinical research results [62]. Some models discuss the relationship between the risk of lymph node metastasis and tumor-specific factors, such as tumor size, grade, depth of invasion, degree of differentiation, and lymphatic invasion, which are closely related [14, 16–21, 25, 28, 30–38, 40–44, 48–50, 52, 54, 63–65]. According to most studies, larger tumors, submucosal invasion, low differentiation, and ulcerative tumors are more likely to undergo lymph node metastasis, which is consistent with previous research conclusions [66]. Lymphatic invasion and tumor invasion depth are established risk factors for lymph node metastasis in early gastric cancer, potentially linked to the prevalence of lymphatics in the gastric proper and submucosal layers [67]. These risk factors have long been discovered and included in surgical assessments, and when patients have the above risk factors, subtotal gastrectomy or lymph node dissection should be considered. However, traditional predictive factors have low specificity and sensitivity and have not yet formed a complete and reliable standard, so there are limitations in their use.

Several models have incorporated novel predictive factors, particularly the assessment of gastric cancer lymph node metastasis risk utilizing imaging data. Zeng et al. [24] focused on enhanced CT images, manually segmenting the lesion images and importing relevant clinical feature data into deep learning networks to form a comprehensive predictive model, comparing the accuracy of different tools, and ultimately forming an accurate and effective risk prediction model with an AUC of 0.901 (95% CI: 0.847–0.956).

Some studies have proposed and evaluated new markers. Dong et al. [22] used whole-genome expression profile data of long non-coding RNA (lncRNA) in primary gastric cancer from the Cancer Genome Atlas (TCGA) to establish a predictive model. By identifying lncRNA expression characteristics in sample data where gastric cancer (GC) occurred with lymph node (LN) metastasis, they found 10 characteristic lncRNA features, including 5 lncRNAs that are relatively highly expressed in lymph node metastasis samples—H19, CECR7, HOTAIR, FAM66D, C22orf34—and 5 lncRNAs that are relatively lowly expressed—TTTY15, TTTY14, TP53TG1, HAR1A, C10orf95. Subsequently, based on deep learning technology, they established a 10-lncRNA risk prediction model and assessed its performance in diagnosing LN metastasis using dataset data and clinical data, finding that its predictive ability was better than traditional markers and CT imaging. Ma et al. [39] also used the TCGA database to identify four miRNAs,including miR-153-3p, miR-708, miR-940, and miR-375—and conducted independent internal and external testing, providing us with new pathological indicators. Long non-coding RNAs (lncRNAs) can be detected in easily accessible body fluids. Compared with traditional endoscopic examinations (which are invasive and have low patient acceptance), sample collection for such biomarkers is more convenient, theoretically enhancing the feasibility of their clinical application and making them suitable for large-scale screening [68]. Meanwhile, lncRNAs are expected to serve as targets for precision therapy in gastric cancer, offering new directions for improving chemotherapy efficacy and overcoming drug resistance [69].

However, research on long non-coding RNAs (lncRNAs) and circular RNAs (circRNAs) is still in its preliminary stage, with detection methods remaining unstandardized and issues of insufficient standardization persisting. And qPCR-based detection is relatively costly and requires specialized equipment, while standardization of results across laboratories remains problematic.

In addition, some studies have proposed new predictive factors, such as the "platelet-to-lymphocyte ratio (PLR)" proposed by Lou et al. [44] the new biomarker "CD44v6" proposed by Suzuki et al. [51] and "UDP-N-acetylglucosamine 2-epimerase/N-acetylmannosamine kinase (GNE)" proposed by Guo et al. [27] According to their research, these new predictive factors have higher specificity and accuracy than original clinical indicators and have clinical application value, but their reliability and applicability still need further research.

Lymph node metastasis risk assessment has great value in the treatment of EGC. Although there is an urgent need for more accurate risk prediction models, we still need more clinical trials to evaluate the performance of various predictive models in order to provide more precise services and better medical care for patients.

### Limitations

It is essential to take into account several limitations. First, the predominance of studies from mainland China may restrict the external validity of findings for other demographic groups, such as European or North American populations. Most models may be difficult to use in other regions or may require further validation before use. For example, Among Asian populations with EGC, the Lauren classification is predominantly of the intestinal type. In contrast, the proportion of the diffuse type is relatively higher in EGC cases in Europe and America, which may be related to differences in genetic susceptibility. The proportion of EGC patients with a family history in Asia (approximately 8%−12%) is higher than that in Europe and America (5%−7%).

In the process of literature inclusion in this study, only English and Chinese studies were included. This language restriction may have led to the exclusion of relevant studies in some non-English and non-Chinese languages (e.g., Japanese, Korean, Spanish, etc.) from the analysis. These excluded studies may contain important information such as unique epidemiological data of early gastric cancer (EGC) in specific regions (e.g., non-English and non-Chinese speaking areas in East Asia with a high incidence of gastric cancer or Spanish-speaking countries), region-specific clinical practice experiences, or associations with genetic factors. As a result, the analysis of EGC differences across regions in this study may be less comprehensive, which to some extent limits the representativeness and generalizability of the study results on a global scale. Additionally, it may introduce potential language-related publication bias, affecting the comprehensive understanding of geographical differences in EGC.

Systematic validation (including internal, external, multicenter, and long-term follow-up validation) is the core to ensure the scientificity, reliability, and clinical value of models, and a key link in promoting precision medicine from theory to practice. In the future, further external validation (especially multicenter and cross-population validation) is needed to test the model's applicability in different ethnicities, medical settings, and sample characteristics, avoid bias from single-center data, and ensure the model has broad promotional value. Secondly, due to varying inclusion criteria and differences in transparency and methodology among the literature, the number of models included in this paper is relatively small. This may not be conducive to further research on the heterogeneity between models and may hinder a better assessment of publication bias. Future evaluations of models will require more rigorous methodologies and enhanced transparency in reporting. This paper is limited to Chinese and English literature, omitting the inclusion and evaluation of works in other languages.

## Conclusion

This systematic review encompassed 50 studies, and the analysis results indicate that the overall c-statistic for the training models was 0.85 [95% CI (0.81–0.89)], suggesting a certain level of predictive ability. All studies included were evaluated as having a high risk of bias, in accordance with the PROBAST checklist standards. Researchers have to strengthen their awareness of and comply with the PROBAST checklist when designing experiments to improve the quality of models in future studies. Future risk assessment studies need to prioritize the establishment of models that utilize larger sample sizes, implement more rigorous study designs, and encompass multicenter sample inclusions in conjunction with external validations.

## Supplementary Information

Supplementary Material 1.

Supplementary Material 1.

Supplementary Material 3.

## Data Availability

All data generated or analysed during this study are included in this published article.

## References

[1] Na JE, Lee YC, Kim TJ, Lee H, Won HH, Min YW, et al. Machine learning model to stratify the risk of lymph node metastasis for early gastric cancer: a single-center cohort study. Cancers (Basel). 2022;14(5):1121. 10.3390/cancers14051121. 35267429 10.3390/cancers14051121 PMC8909118

[2] Han B, Zheng R, Zeng H, Wang S, Sun K, Chen R, et al. Cancer incidence and mortality in China, 2022. J Natl Cancer Center. 2024;4(1):47–53. 10.1016/j.jncc.2024.01.006. 39036382 10.1016/j.jncc.2024.01.006 PMC11256708

[3] Waddingham W, Nieuwenburg SAV, Carlson S, Rodriguez-Justo M, Spaander M, Kuipers EJ, et al. Recent advances in the detection and management of early gastric cancer and its precursors. Frontline Gastroenterol. 2020;12(4):322–31. 10.1136/flgastro-2018-101089. 34249318 10.1136/flgastro-2018-101089 PMC8223672

[4] Miao R, Li ZY, Ji JF. Analysis of the current status and development trend of diagnosis and treatment of early gastric cancer in China based on relevant data from the China Gastrointestinal Oncology Surgery Alliance. Chin J Pract Surg. 10.19538/j.cjps.issn1005-2208.2019.05.03.

[5] Wang YY, Wang TX, Yang B, Ma CZ, Wang X, Li YM. Establishment and validation of a prognostic model for gastric cancer patients after radical resection based on metastatic lymph node ratio. J Lanzhou Univ (Med Sci). 2023;49(8):40–46. 10.13885/j.issn.1000-2812.2023.08.006.

[6] Soetikno R, Kaltenbach T, Yeh R, Gotoda T. Endoscopic mucosal resection for early cancers of the upper gastrointestinal tract. J Clin Oncol. 2005;23(20):4490–8. 10.1200/JCO.2005.19.935. 16002839 10.1200/JCO.2005.19.935

[7] Japanese Gastric Cancer Association. Japanese Gastric Cancer Treatment Guidelines 2021 (6th edition). Gastric Cancer. 2023;26(1):1–25. 10.1007/s10120-022-01331-8. 10.1007/s10120-022-01331-8 PMC9813208 36342574

[8] Isomoto H, Shikuwa S, Yamaguchi N, Fukuda E, Ikeda K, Nishiyama H, et al. Endoscopic submucosal dissection for early gastric cancer: a large-scale feasibility study. Gut. 2009;58(3):331–6. 10.1136/gut.2008.165381. 19001058 10.1136/gut.2008.165381

[9] Moons KG, de Groot JA, Bouwmeester W, Vergouwe Y, Mallett S, Altman DG, et al. Critical appraisal and data extraction for systematic reviews of prediction modelling studies: the CHARMS checklist. PLoS Med. 2014;11(10):e1001744. 10.1371/journal.pmed.1001744. 25314315 10.1371/journal.pmed.1001744 PMC4196729

[10] Guyatt GH, Oxman AD, Vist GE, Kunz R, Falck-Ytter Y, Alonso-Coello P, et al. GRADE: an emerging consensus on rating quality of evidence and strength of recommendations. BMJ. 2008;336(7650):924–6. 10.1136/bmj.39489.470347. 18436948 10.1136/bmj.39489.470347.AD PMC2335261

[11] Moons KGM, Wolff RF, Riley RD, Whiting PF, Westwood M, Collins GS, et al. PROBAST: A Tool to Assess Risk of Bias and Applicability of Prediction Model Studies: Explanation and Elaboration. Ann Intern Med. 2019;170(1):W1–33. 10.7326/M18-1377. 30596876 10.7326/M18-1377

[12] Higgins JP, Thompson SG, Deeks JJ, Altman DG. Measuring inconsistency in meta-analyses. BMJ. 2003;327(7414):557–60. 10.1136/bmj.327.7414.557. 12958120 10.1136/bmj.327.7414.557 PMC192859

[13] Egger M, Davey Smith G, Schneider M, Minder C. Bias in meta-analysis detected by a simple, graphical test. BMJ. 1997;315(7109):629–34. 10.1136/bmj.315.7109.629. 9310563 10.1136/bmj.315.7109.629 PMC2127453

[14] Wang X, Yang X, Cai F, Cai M, Liu Y, Zhang L, et al. The key role of tumor budding in predicting the status of lymph node involvement in early gastric cancer patients: a clinical multicenter validation in China. Ann Surg Oncol. 2024;31(7):4224–35. 10.1245/s10434-024-15229-5. 38536585 10.1245/s10434-024-15229-5

[15] Zhao L, Han W, Niu P, Lu Y, Zhang F, Jiao F, et al. Using nomogram, decision tree, and deep learning models to predict lymph node metastasis in patients with early gastric cancer: a multi-cohort study. Am J Cancer Res. 2023;13(1):204–15. 36777507 PMC9906085

[16] You H, Chen S, Wang S. A nomogram for predicting lymph node metastasis in early gastric signet ring cell carcinoma. Sci Rep. 2023;13(1):15039. 10.1038/s41598-023-40733-1. 37699908 10.1038/s41598-023-40733-1 PMC10497562

[17] Yang JJ, Wang XY, Ma R, Chen MH, Zhang GX, Li X. Prediction of lymph node metastasis in early gastric signet-ring cell carcinoma: a real-world retrospective cohort study. World J Gastroenterol. 2023;29(24):3807–24. 10.3748/wjg.v29.i24.3807. 37426318 10.3748/wjg.v29.i24.3807 PMC10324532

[18] Wu H, Liu W, Yin M, Liu L, Qu S, Xu W, et al. A nomogram based on platelet-to-lymphocyte ratio for predicting lymph node metastasis in patients with early gastric cancer. Front Oncol. 2023;13:1201499. 10.3389/fonc.2023.1201499. 37719022 10.3389/fonc.2023.1201499 PMC10502215

[19] Li J, Cui T, Huang Z, Mu Y, Yao Y, Xu W, et al. Analysis of risk factors for lymph node metastasis and prognosis study in patients with early gastric cancer: a SEER data-based study. Front Oncol. 2023;13:1062142. 10.3389/fonc.2023.1062142. 37007147 10.3389/fonc.2023.1062142 PMC10064290

[20] Li C, Xie S, Chen D, Zhang J, Zhang N, Mu J, et al. Clinicopathological characteristics of early gastric cancer with different level of undifferentiated component and nomogram to predict lymph node metastasis. Front Surg. 2023;10:1097927. 10.3389/fsurg.2023.1097927. 36865628 10.3389/fsurg.2023.1097927 PMC9972584

[21] Lee HD, Nam KH, Shin CM, Lee HS, Chang YH, Yoon H, et al. Development and validation of models to predict lymph node metastasis in early gastric cancer using logistic regression and gradient boosting machine methods. Cancer Res Treat. 2023;55(4):1240–9. 10.4143/crt.2022.1330. 36960625 10.4143/crt.2022.1330 PMC10582533

[22] Dong ZB, Xiang HT, Wu HM, Cai XL, Chen ZW, Chen SS, et al. LncRNA expression signature identified using genome-wide transcriptomic profiling to predict lymph node metastasis in patients with stage T1 and T2 gastric cancer. Gastric Cancer. 2023;26(6):947–57. 10.1007/s10120-023-01428-8. 37691031 10.1007/s10120-023-01428-8 PMC10640531

[23] Zhu H, Wang G, Zheng J, Zhu H, Huang J, Luo E, et al. Preoperative prediction for lymph node metastasis in early gastric cancer by interpretable machine learning models: a multicenter study. Surgery. 2022;171(6):1543–51. 10.1016/j.surg.2021.12.015. 35131110 10.1016/j.surg.2021.12.015

[24] Zeng Q, Li H, Zhu Y, Feng Z, Shu X, Wu A, et al. Development and validation of a predictive model combining clinical, radiomics, and deep transfer learning features for lymph node metastasis in early gastric cancer. Front Med. 2022;9:986437. 10.3389/fmed.2022.986437. 10.3389/fmed.2022.986437 PMC9573999 36262277

[25] Yang T, Martinez-Useros J, Liu J, Alarcón I, Li C, Li W, et al. A retrospective analysis based on multiple machine learning models to predict lymph node metastasis in early gastric cancer. Front Oncol. 2022;12:1023110. 10.3389/fonc.2022.1023110. 36530978 10.3389/fonc.2022.1023110 PMC9751349

[26] Li X, Zhou H, Zhao X, Peng H, Luo S, Feng J, et al. Establishment and validation for predicting the lymph node metastasis in early gastric adenocarcinoma. J Healthc Eng. 2022;2022:8399822. 10.1155/2022/8399822. 35812896 10.1155/2022/8399822 PMC9259240

[27] Guo X, Gu J, Xue A, Song S, Liu B, Gao X, et al. Loss of GNE predicts lymph node metastasis in early gastric cancer. Cells. 2022;11(22):3624. 10.3390/cells11223624. 36429052 10.3390/cells11223624 PMC9688572

[28] Cai F, Dong Y, Wang P, Zhang L, Yang Y, Liu Y, et al. Risk assessment of lymph node metastasis in early gastric cancer: establishment and validation of a seven-point scoring model. Surgery. 2022;171(5):1273–80. 10.1016/j.surg.2021.10.049. 34865863 10.1016/j.surg.2021.10.049

[29] Zhou CM, Wang Y, Ye HT, Yan S, Ji M, Liu P, et al. Machine learning predicts lymph node metastasis of poorly differentiated-type intramucosal gastric cancer. Sci Rep. 2021;11(1):1300. 10.1038/s41598-020-80582-w. 33446730 10.1038/s41598-020-80582-w PMC7809018

[30] Zhang M, Ding C, Xu L, Feng S, Ling Y, Guo J, et al. A nomogram to predict risk of lymph node metastasis in early gastric cancer. Sci Rep. 2021;11(1):22873. 10.1038/s41598-021-02305-z. 34819570 10.1038/s41598-021-02305-z PMC8613278

[31] Wei J, Zhang Y, Liu Y, Wang A, Fan B, Fu T, et al. Construction and validation of a risk-scoring model that preoperatively predicts lymph node metastasis in early gastric cancer patients. Ann Surg Oncol. 2021;28(11):6665–72. 10.1245/s10434-021-09867-2. 33783640 10.1245/s10434-021-09867-2

[32] Wang J, Wang L, Li S, Bai F, Xie H, Shan H, et al. Risk factors of lymph node metastasis and its prognostic significance in early gastric cancer: a multicenter study. Front Oncol. 2021;11:649035. 10.3389/fonc.2021.649035. 34722232 10.3389/fonc.2021.649035 PMC8548692

[33] Sui W, Chen Z, Li C, Chen P, Song K, Wei Z, et al. Nomograms for Predicting the Lymph Node Metastasis in Early Gastric Cancer by Gender: A Retrospective Multicentric Study. Front Oncol. 2021;29(11):616951. 10.3389/fonc.2021.616951. 10.3389/fonc.2021.616951 PMC8511824 34660252

[34] Pan S, An W, Tan Y, Chen Q, Liu P, Xu H. Prediction model of lymph node metastasis risk in elderly patients with early gastric cancer before endoscopic resection: a retrospective analysis based on international multicenter data. J Cancer. 2021;12(18):5583–92. 10.7150/jca.56702. 34405019 10.7150/jca.56702 PMC8364644

[35] Mei Y, Wang S, Feng T, Yan M, Yuan F, Zhu Z, et al. Nomograms involving HER2 for predicting lymph node metastasis in early gastric cancer. Front Cell Dev Biol. 2021;9:781824. 10.3389/fcell.2021.781824. 35004681 10.3389/fcell.2021.781824 PMC8740268

[36] Jin F, Qian X, Ni F, Pan SY. Risk factors and risk model of lymph node metastasis in early gastric cancer]. Zhonghua Yu Fang Yi Xue Za Zhi. 2021;55(8):990–4. 10.3760/cma.j.cn112150-20200805-01095. Chinese. 34445838 10.3760/cma.j.cn112150-20200805-01095

[37] Yin XY, Pang T, Liu Y, Cui HT, Luo TH, Lu ZM, et al. Development and validation of a nomogram for preoperative prediction of lymph node metastasis in early gastric cancer. World J Surg Oncol. 2020;18(1):2. 10.1186/s12957-019-1778-2. 31898548 10.1186/s12957-019-1778-2 PMC6941310

[38] Kim SM, Min BH, Ahn JH, Jung SH, An JY, Choi MG, et al. Nomogram to predict lymph node metastasis in patients with early gastric cancer: a useful clinical tool to reduce gastrectomy after endoscopic resection. Endoscopy. 2020;52(6):435–43. 10.1055/a-1117-3059. 32162286 10.1055/a-1117-3059

[39] Ma M, Lu S, Liu Y, Kong P, Long Z, Wan P, et al. Identification and external validation of a novel miRNA signature for lymph node metastasis prediction in submucosal-invasive gastric cancer patients. Cancer Med. 2019;8(14):6315–63. 10.1002/cam4.2530.25. 31486298 10.1002/cam4.2530 PMC6797584

[40] Lin JX, Wang ZK, Wang W, Desiderio J, Xie JW, Wang JB, et al. Risk factors of lymph node metastasis or lymphovascular invasion for early gastric cancer: a practical and effective predictive model based on international multicenter data. BMC Cancer. 2019;19(1):1048. 10.1186/s12885-019-6147-6. 31694573 10.1186/s12885-019-6147-6 PMC6836519

[41] Chen D, Chen G, Jiang W, Fu M, Liu W, Sui J, et al. Association of the collagen signature in the tumor microenvironment with lymph node metastasis in early gastric cancer. JAMA Surg. 2019;154(3):e185249. 10.1001/jamasurg.2018.5249. 30698615 10.1001/jamasurg.2018.5249 PMC6439641

[42] Zhang Y, Liu Y, Zhang J, Wu X, Ji X, Fu T, et al. Construction and external validation of a nomogram that predicts lymph node metastasis in early gastric cancer patients using preoperative parameters. Chin J Cancer Res. 2018;30(6):623–32. 10.21147/j.issn.1000-9604.2018.06.07. 30700931 10.21147/j.issn.1000-9604.2018.06.07 PMC6328510

[43] Gu L, Chen M, Khadaroo PA, Ma X, Kong L, Li X, et al. A risk-scoring model for predicting lymph node metastasis in early gastric cancer patients: a retrospective study and external validation. J Gastrointest Surg. 2018;22(9):1508–15. 10.1007/s11605-018-3816-8. 29845571 10.1007/s11605-018-3816-8

[44] Lou N, Zhang L, Chen XD, Pang WY, Arvine C, Huang YP, et al. A novel scoring system associating with preoperative platelet/lymphocyte and clinicopathologic features to predict lymph node metastasis in early gastric cancer. J Surg Res. 2017;209:153–61. 10.1016/j.jss.2016.10.011. 28032552 10.1016/j.jss.2016.10.011

[45] Guo CG, Zhao DB, Liu Q, Zhou ZX, Zhao P, Wang GQ, et al. A nomogram to predict lymph node metastasis in patients with early gastric cancer. Oncotarget. 2017;8(7):12203–10. 10.18632/oncotarget.14660. 28099943 10.18632/oncotarget.14660 PMC5355337

[46] Zheng Z, Zhang Y, Zhang L, Li Z, Wu X, Liu Y, et al. A nomogram for predicting the likelihood of lymph node metastasis in early gastric patients. BMC Cancer. 2016;16(1):92. 10.1186/s12885-016-2132-5. 26873736 10.1186/s12885-016-2132-5 PMC4751748

[47] Zhao LY, Yin Y, Li X, Zhu CJ, Wang YG, Chen XL, et al. A nomogram composed of clinicopathologic features and preoperative serum tumor markers to predict lymph node metastasis in early gastric cancer patients. Oncotarget. 2016;7(37):59630–9. 10.18632/oncotarget.10732. 27449100 10.18632/oncotarget.10732 PMC5312336

[48] Sekiguchi M, Oda I, Taniguchi H, Suzuki H, Morita S, Fukagawa T, et al. Risk stratification and predictive risk-scoring model for lymph node metastasis in early gastric cancer. J Gastroenterol. 2016;51(10):961–70. 10.1007/s00535-016-1180-6. 26884381 10.1007/s00535-016-1180-6

[49] Pyo JH, Shin CM, Lee H, Min BH, Lee JH, Kim SM, et al. A risk-prediction model based on lymph-node metastasis for incorporation into a treatment algorithm for signet ring cell-type intramucosal gastric cancer. Ann Surg. 2016;264(6):1038–43. 10.1097/SLA.0000000000001602. 27828821 10.1097/SLA.0000000000001602

[50] Guo CG, Chen YJ, Ren H, Zhou H, Shi JF, Yuan XH, et al. A nomogram for predicting the likelihood of lymph node metastasis in early gastric signet ring cell carcinoma: a single center retrospective analysis with external validation. Medicine (Baltimore). 2016;95(46):e5393. 10.1097/MD.0000000000005393. 27861374 10.1097/MD.0000000000005393 PMC5120931

[51] Eom BW, Joo J, Park B, Jo MJ, Choi SH, Cho SJ, et al. Nomogram incorporating CD44v6 and clinicopathological factors to predict lymph node metastasis for early gastric cancer. PLoS One. 2016;11(8):e0159424. 10.1371/journal.pone.0159424. 27482895 10.1371/journal.pone.0159424 PMC4970798

[52] Zheng Z, Zhang Y, Zhang L, Li Z, Wu A, Wu X, et al. Nomogram for predicting lymph node metastasis rate of submucosal gastric cancer by analyzing clinicopathological characteristics associated with lymph node metastasis. Chin J Cancer Res. 2015;27(6):572–9. 10.3978/j.issn.1000-9604.2015.12.06. 26752931 10.3978/j.issn.1000-9604.2015.12.06 PMC4697108

[53] Jiang XC, Yao XB, Xia HB, Su YZ, Luo PQ, Sun JR, et al. Nomogram established using risk factors of early gastric cancer for predicting the lymph node metastasis. World J Gastrointest Oncol. 2023;15(4):665–76. 10.4251/wjgo.v15.i4.665. 37123061 10.4251/wjgo.v15.i4.665 PMC10134212

[54] So S, Noh JH, Ahn JY, Lee IS, Lee JB, Jung HY, et al. Scoring model based on nodal metastasis prediction suggesting an alternative treatment to total gastrectomy in proximal early gastric cancer. J Gastric Cancer. 2022;22(1):24–34. 10.5230/jgc.2022.22.e3. 35425656 10.5230/jgc.2022.22.e3 PMC8980596

[55] Bray F, Laversanne M, Sung H, Ferlay J, Siegel RL, Soerjomataram I, et al. Global cancer statistics 2022: GLOBOCAN estimates of incidence and mortality worldwide for 36 cancers in 185 countries. CA Cancer J Clin. 2024;74(3):229–63. 10.3322/caac.21834. 38572751 10.3322/caac.21834

[56] Smyth EC, Nilsson M, Grabsch HI, van Grieken NC, Lordick F. Gastric cancer. Lancet. 2020;29(10251):635–48. 10.1016/S0140-6736(20)31288-5. 10.1016/S0140-6736(20)31288-5 32861308

[57] Ishikawa S, Togashi A, Inoue M, Honda S, Nozawa F, Toyama E, et al. Indications for EMR/ESD in cases of early gastric cancer: relationship between histological type, depth of wall invasion, and lymph node metastasis. Gastric Cancer. 2007;10(1):35–8. 10.1007/s10120-006-0407-2. 17334716 10.1007/s10120-006-0407-2

[58] Pessorrusso FCS, Felipe-Silva A, Jacob CE, Ramos MFKP, Ferreira VAA, de Mello ES, et al. Risk assessment of lymph node metastases in early gastric adenocarcinoma fulfilling expanded endoscopic resection criteria. Gastrointest Endosc. 2018;88(6):912–8. 10.1016/j.gie.2018.07.023. 30053392 10.1016/j.gie.2018.07.023

[59] Wang Z, Li H, Carpenter C, Guan Y. Challenge-enabled machine learning to drug-response prediction. AAPS J. 2020;22(5):106. 10.1208/s12248-020-00494-5. 32778984 10.1208/s12248-020-00494-5 PMC10176199

[60] Serghiou S, Rough K. Deep learning for epidemiologists: an introduction to neural networks. Am J Epidemiol. 2023;192(3):1904–16. 10.1093/aje/kwad107. 37139570 10.1093/aje/kwad107 PMC13368595

[61] Li Y, Xie F, Xiong Q, Lei H, Feng P. Machine learning for lymph node metastasis prediction of in patients with gastric cancer: a systematic review and meta-analysis. Front Oncol. 2022;12:946038. 10.3389/fonc.2022.946038. 36059703 10.3389/fonc.2022.946038 PMC9433672

[62] Wang ZK, Lin JX, Li P, Xie JW, Wang JB, Lu J, et al. Higher risk of lymph node metastasis in young patients with early gastric cancer. J Cancer. 2019;10(18):4389–96. 10.7150/jca.30260. 31413759 10.7150/jca.30260 PMC6691700

[63] Yoo HJ, Lee H, Lee HH, Lee JH, Jun KH, Kim JJ, et al. A nomogram for predicting extraperigastric lymph node metastasis in patients with early gastric cancer. J Gastric Cancer. 2023;23(2):355–64. 10.5230/jgc.2023.23.e18. 37129158 10.5230/jgc.2023.23.e18 PMC10154132

[64] Tian H, Ning Z, Zong Z, Liu J, Hu C, Ying H, et al. Application of machine learning algorithms to predict lymph node metastasis in early gastric cancer. Front Med. 2022;8:759013. 10.3389/fmed.2021.759013. 10.3389/fmed.2021.759013 PMC8806156 35118083

[65] Cui H, Cao B, Deng H, Liu GB, Liang WQ, Xie TY, et al. A nomogram for predicting lymph node metastasis in early gastric cancer. Zhonghua Wei Chang Wai Ke Za Zhi. 2022;25(1):40–7. 10.3760/cma.j.cn441530-20210208-00059. 35067033 10.3760/cma.j.cn441530-20210208-00059

[66] Oh YJ, Kim DH, Han WH, Eom BW, Kim YI, Yoon HM, et al. Risk factors for lymph node metastasis in early gastric cancer without lymphatic invasion after endoscopic submucosal dissection. Eur J Surg Oncol. 2021;47(12):3059–63. 10.1016/j.ejso. 33934939 10.1016/j.ejso.2021.04.029

[67] Li X, Liu S, Yan J, et al. The characteristics, prognosis, and risk factors of lymph node metastasis in early gastric cancer. Gastroenterol Res Pract. 2018;2018:6945743. 10.1155/2018/6945743. 29853864 10.1155/2018/6945743 PMC5954923

[68] Necula L, Matei L, Dragu D, Neagu AI, Mambet C, Nedeianu S, et al. Recent advances in gastric cancer early diagnosis. World J Gastroenterol. 2019;25(17):2029–44. 10.3748/wjg.v25.i17.2029. 31114131 10.3748/wjg.v25.i17.2029 PMC6506585

[69] Yuan L, Xu ZY, Ruan SM, Mo S, Qin JJ, Cheng XD. Long non-coding RNAs towards precision medicine in gastric cancer: early diagnosis, treatment, and drug resistance. Mol Cancer. 2020;19(1):96. 10.1186/s12943-020-01219-0. 32460771 10.1186/s12943-020-01219-0 PMC7251695

[70] Kim S, Bae WJ, Ahn JM, Heo JH, Kim KM, Choi KW, et al. Microrna signatures associated with lymph node metastasis in intramucosal gastric cancer. Mod Pathol. 2021;34(3):672–83. 10.1038/s41379-020-00681-x. 32973329 10.1038/s41379-020-00681-x

