# Supplementary figures and images for: Risk prediction models for lymph node metastasis in early gastric cancer patients: a systematic review and meta-analysis
Source: BMC Gastroenterol. 2025 Oct 31;25:776. doi: 10.1186/s12876-025-04342-8 (PMC12577095; doi:10.1186/s12876-025-04342-8)

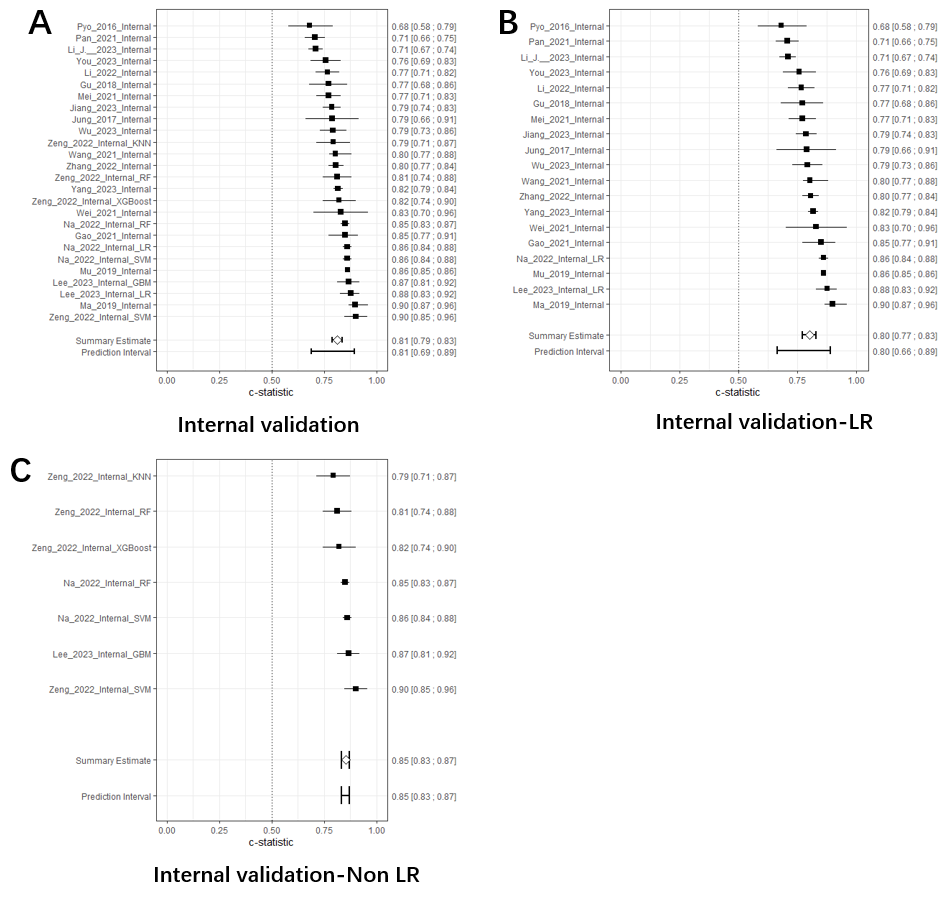

Supplement: Supplementary file 3 — Supplementary Material 3. [file 12876_2025_4342_MOESM3_ESM.zip › Internal validation.tiff]

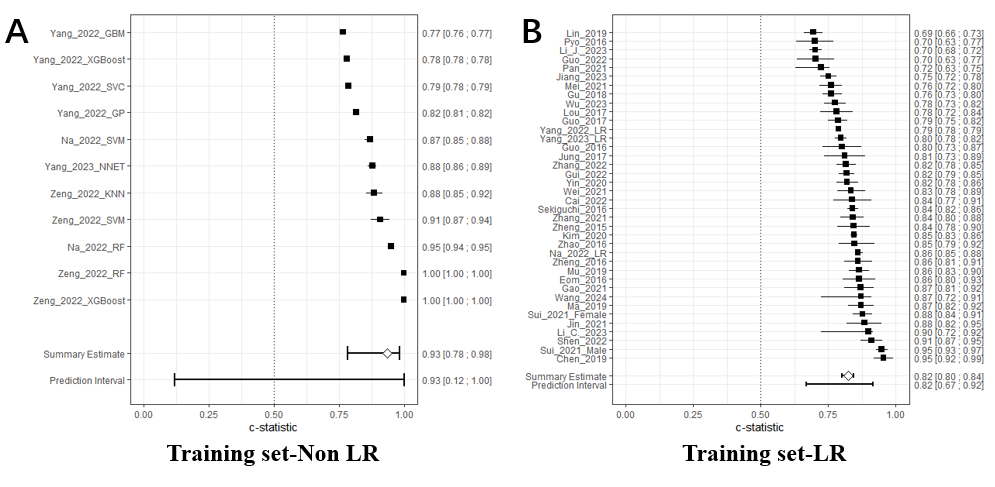

Supplement: Supplementary file 3 — Supplementary Material 3. [file 12876_2025_4342_MOESM3_ESM.zip › Training set.tiff]

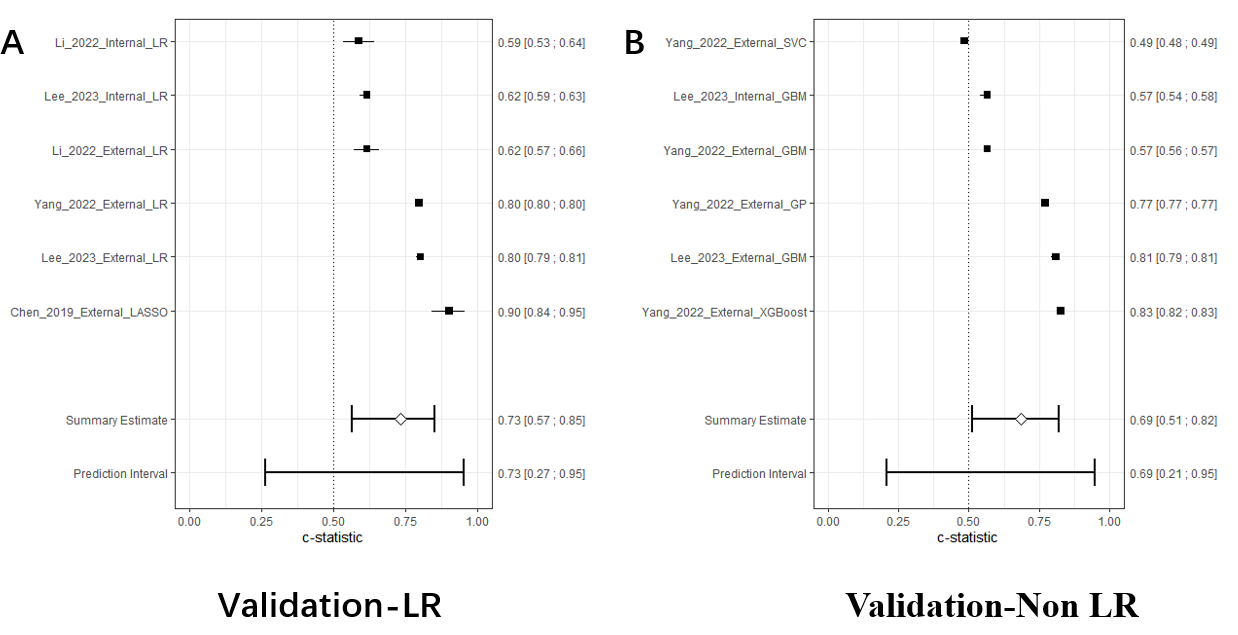

Supplement: Supplementary file 3 — Supplementary Material 3. [file 12876_2025_4342_MOESM3_ESM.zip › Validation.tiff]

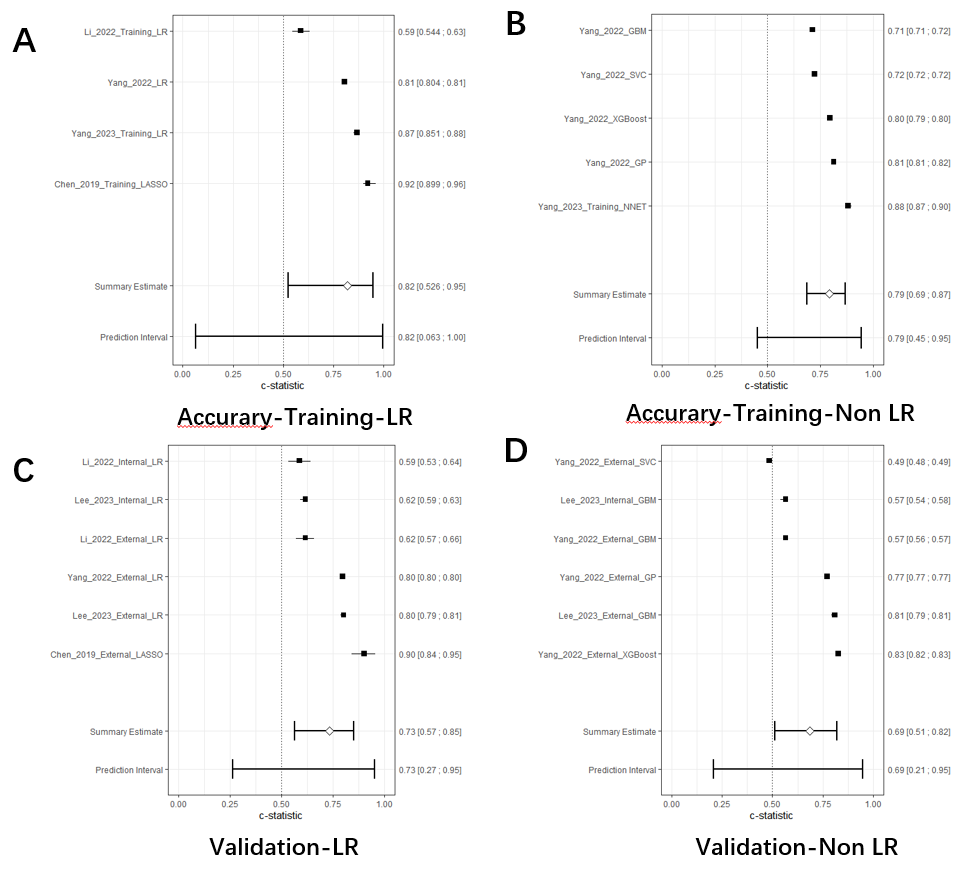

Supplement: Supplementary file 3 — Supplementary Material 3. [file 12876_2025_4342_MOESM3_ESM.zip › Accurary.tiff]

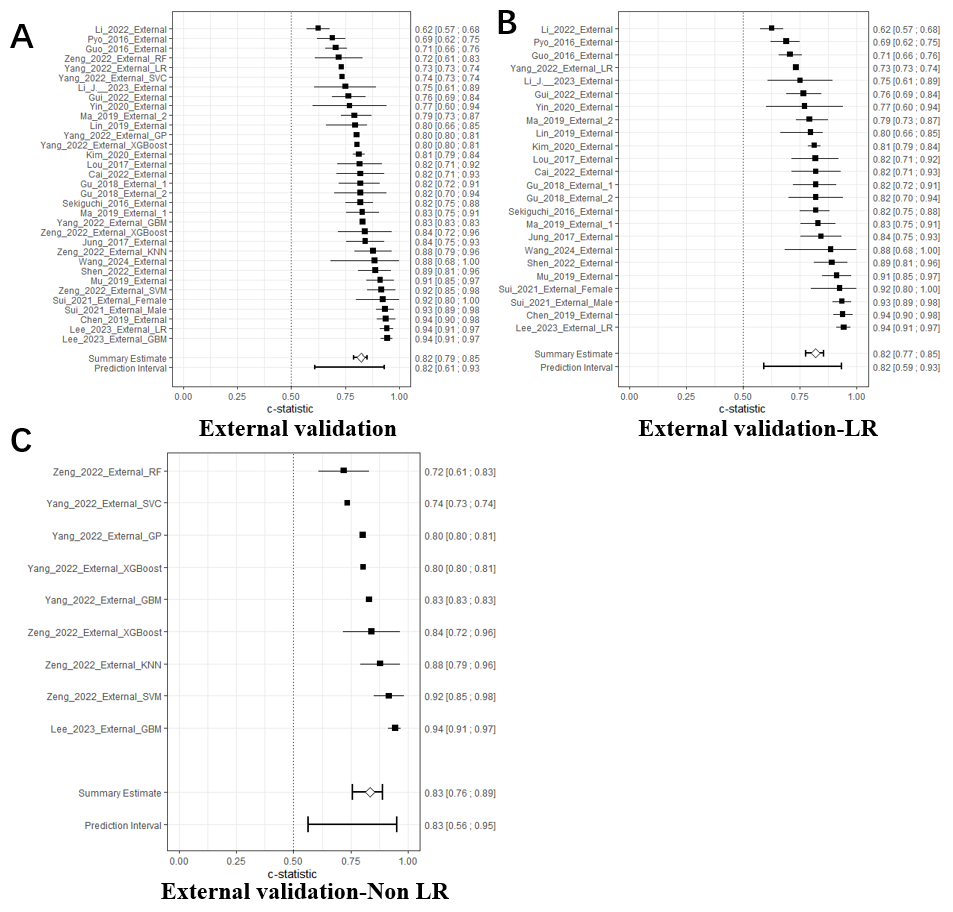

Supplement: Supplementary file 3 — Supplementary Material 3. [file 12876_2025_4342_MOESM3_ESM.zip › External validation.tiff]

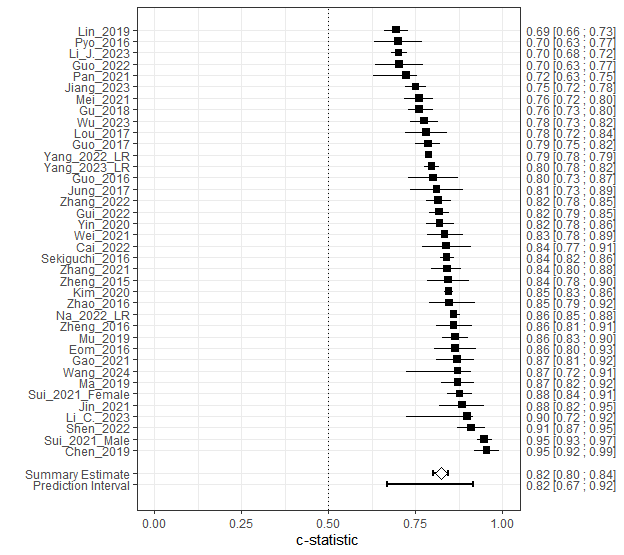

Supplement: Supplementary file 3 — Supplementary Material 3. [file 12876_2025_4342_MOESM3_ESM.zip › Forest-Training set-LR.tiff]

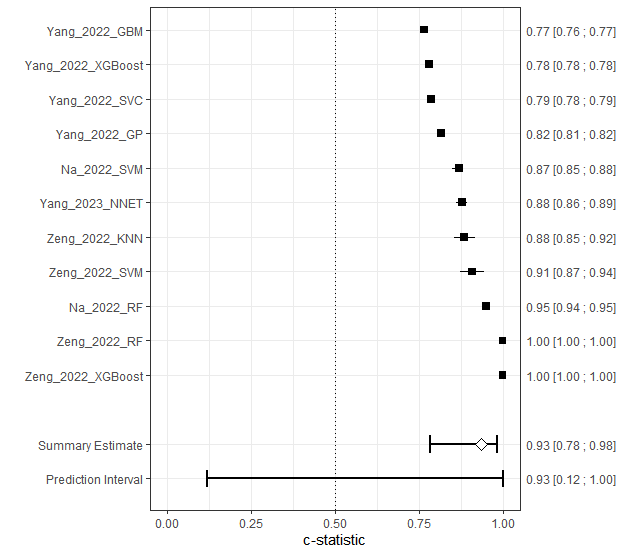

Supplement: Supplementary file 3 — Supplementary Material 3. [file 12876_2025_4342_MOESM3_ESM.zip › Forest-Training set-Non LR.tiff]
